# Genetic Incompatibilities Between Mitochondria and Nuclear Genes: Effect on Gene Flow and Speciation

**DOI:** 10.3389/fgene.2019.00062

**Published:** 2019-02-13

**Authors:** Arndt Telschow, Jürgen Gadau, John H. Werren, Yutaka Kobayashi

**Affiliations:** ^1^Institute for Environmental Systems Research, Osnabrück University, Osnabrück, Germany; ^2^Institute for Evolution and Biodiversity, Westfalian Wilhelms-University, Münster, Germany; ^3^Department of Biology, University of Rochester, Rochester, NY, United States; ^4^School of Economics and Management, Kochi University of Technology, Kami, Japan

**Keywords:** gene flow, mathematical model, speciation, nuclear-cytoplasmic incompatibility, mitochondria, effective migration rate, fitness graph

## Abstract

The process of speciation is, according to the biological species concept, the reduction in gene flow between genetically diverging populations. Most of the previous theoretical studies analyzed the effect of nuclear genetic incompatibilities on gene flow. There is, however, an increasing number of empirical examples suggesting that cytoplasmically inherited genetic elements play an important role in speciation. Here, we present a theoretical analysis of mitochondrial driven speciation, in which genetic incompatibilities occur between mitochondrial haplotypes and nuclear alleles. Four population genetic models with mainland-island structure were analyzed that differ with respect to the type of incompatibility and the underlying genetics. Gene flow reduction was measured on selectively neutral alleles of an unlinked locus and quantified by the effective migration rate. Analytical formulae for the different scenarios were derived using the fitness graph method. For the models with haploid genetics, we found that mito-nuclear incompatibilities (MtNI) are as strong as nuclear-nuclear incompatibilities (NNI) in reducing gene flow at the unlinked locus, but only if males and females migrate in equal number. For models with diploid genetics, we found that MtNI reduce gene flow stronger than NNI when incompatibilities are recessive, but weaker when they are dominant. For both haploid and diploid MtNI, we found that gene flow reduction is stronger if females are the migrating sex, but weaker than NNI when males are the migrating sex. These results encourage further examination on the role of mitochondria on genetic divergence and speciation and point toward specific factors (e.g., migrating sex) that could be the focus of an empirical test.

## Introduction

The biological species concept defines species as “groups of actually or potentially interbreeding natural populations that are reproductively isolated from other such groups” (Mayr, 1963; Coyne and Orr, 2004; Gavrilets, 2004). Reproductive isolation is often measured by the degree of genetic exchange between populations (Bengtsson, 1985; Gavrilets, 1997; Kobayashi and Telschow, 2008). In this framework, the process of speciation is considered as the reduction in gene flow between diverging populations. Understanding the mechanisms and factors that cause gene flow reduction is therefore a central question in speciation research.

Most of the previous theoretical studies on reproductive isolation focused on incompatibility mechanisms based on nuclear loci, e.g., Dobzhansky-Muller incompatibility (Bengtsson, 1985; Barton and Bengtsson, 1986; Gavrilets, 1997; Piálek and Barton, 1997; Gavrilets and Cruzan, 1998; Navarro and Barton, 2003). These studies investigated how gene flow is modified at a neutral marker locus in the presence of incompatibility loci. To quantify the gene flow, the *effective migration rate* was used. This concept was first introduced by Bengtsson and Barton to quantify the reduction in gene flow at a genetic cline (Bengtsson, 1985; Barton and Bengtsson, 1986), though its original idea goes back to Nagylaki (1976). Roughly speaking, the effective migration rate is the migration rate, which, if applied to a model with no gene-flow barrier, generates the same allele frequency change as in a focal model with the genetic barrier (Kobayashi et al., 2008). Analogous concepts are the effective population size, which quantifies random genetic drift (see e.g., Crow and Kimura, 1970), and the effective recombination rate, which quantifies recombination intensity (Kobayashi and Telschow, 2011).

The calculation of the effective migration rate is, from a mathematical point of view, an eigenvalue problem (Kobayashi et al., 2008). An important field of application is the analysis of speciation models. Various analytical formulae were derived for gene flow reduction by genetic incompatibilities, including Dobzhansky-Muller incompatibility and cytoplasmic incompatibility (Bengtsson, 1985; Barton and Bengtsson, 1986; Gavrilets, 1997; Piálek and Barton, 1997; Gavrilets and Cruzan, 1998; Telschow et al., 2002, 2007; Navarro and Barton, 2003). The effective migration rate was also used to investigate gene flow enhancement due to heterosis (Ingvarsson and Whitlock, 2000), and gene flow modification caused by cytoplasmically inherited bacteria (Telschow et al., 2006; Engelstädter et al., 2008; Kobayashi and Telschow, 2010; Kobayashi et al., 2011). A major insight of the latter studies is that sex ratio distorters increase the genetic influx to a population and thus impede local adaptation (Telschow et al., 2006; Kobayashi et al., 2011).

Over the last decade it has become clear that nuclear-cytoplasmic incompatibilities (including mitochondria and other plastids) are common and could be important drivers of speciation throughout the tree of life (fungi, plants, and animals; Lee et al., 2008; Gershoni et al., 2009; Chou and Leu, 2010; Meiklejohn et al., 2013). These incompatibilities are a result of the evolutionary history of plastids, i.e., they evolved from obligate endosymbionts, which have over time lost most of their own genome but retained crucial genes for essential functions in the energy metabolism of plants and animals (Rand et al., 2004). However, to perform their function mitochondria need more than 1,000 nuclear encoded genes and some of these nuclear encoded genes interact very closely in large protein complexes of the oxidative phosphorylation system (Gershoni et al., 2009). The final aspect that makes these interactions interesting is the difference in evolutionary rates between plastids and nuclear genes. Plastids evolve or accumulate changes much faster due to their smaller effective population size, strictly maternal inheritance (in most cases) and in some cases higher mutation rates. While substitution rates in nuclear genes are similar in plants and animals (Lynch, 2010), mitochondrial substitution rates are much higher in animals than in plants, and can vary considerably among taxa (Oliveira et al., 2008; Lynch, 2010; Yoshizawa and Johnson, 2013; Cameron, 2014; Li et al., 2017), sometimes over 40 fold (Oliveira et al., 2008). These higher substitution rates are believed to be driven by higher mitochondrial mutation rates.

One of the best-studied system in this respect is the *Nasonia* species complex (Gadau et al., 1999; Niehuis et al., 2008; Koevoets and Beukeboom, 2009; Werren et al., 2010). *Nasonia* is a hymenopteran parasitoid, which parasitizes fly pupae. Nasonia is haplodiploid like all Hymenoptera, i.e., males are haploid and develop from unfertilized eggs whereas females are diploid and develop from fertilized eggs. All *Nasonia* species are infected with *Wolbachia*, which cause cytoplasmic incompatibility. But once individuals are cured from *Wolbachia* they can produce viable and fertile F1 females. F2 hybrid males show a wide range of postzygotic hybrid breakdown traits. The strength of the observed hybrid breakdown depends both on environmental conditions and the direction of the cross, i.e., nuclear-cytoplasm combination (Breeuwer and Werren, 1995; Gadau et al., 1999; Niehuis et al., 2008; Koevoets and Beukeboom, 2009). Introgressions of the mitochondria between the species further supports the role of mitochondria in the nuclear-cytoplasmic incompatibilities (Breeuwer and Werren, 1995).

In the present study, we follow previous theoretical work on nuclear-cytoplasmic disequilibria (Schnabel and Asmussen, 1989; Paige et al., 1991; Arnold, 1993), and analyze the effect of mito-nuclear incompatibilities (MtNI) on gene flow at a neutral marker locus, and how they differ from nuclear-nuclear incompatibilities (NNI). Our study highlights specific factors that might influence the efficiency of nuclear-cytoplasmic incompatibilities in the context of speciation.

In the remainder of this paper, we analyze four mainland-island models, which all assume a gene-flow barrier, but differ in the type of genetic incompatibility and the underlying genetic system. This mimics a situation where a potential nuclear-cytoplasmic incompatibility evolved in a subpopulation and describes the potential of this incompatibility to lead to a complete disruption of gene flow between these two populations. Following previous studies, we compute the effective migration rate for each of the four models and compare the results. We show that dominance and unequal migration rates of males and females play important roles in generating discrepancies between mito-nuclear and nuclear-nuclear incompatibilities, and that the former can generate a stronger barrier to gene flow than the latter depending on parameters. In conclusion, the results generally support the view that mitochondria could promote nuclear genetic divergence and speciation at least as much or even more than nuclear-nuclear incompatibilities.

## Materials and Methods

### Model Structure

We consider a discrete-generation model with two populations of sexually reproducing organisms in a mainland-island spatial context, in which immigrants from the mainland population in every generation replace a constant fraction *m* of the island population. In all scenarios considered below, the original pure strain from the island and that from the mainland are both equally viable and fertile, and we set their relative fitness to one without loss of generality. On the other hand, hybrid strains produced on the island by mating between residents and migrants may suffer from reproductive incompatibility, undergoing reduced fitness.

**Figure 1:**
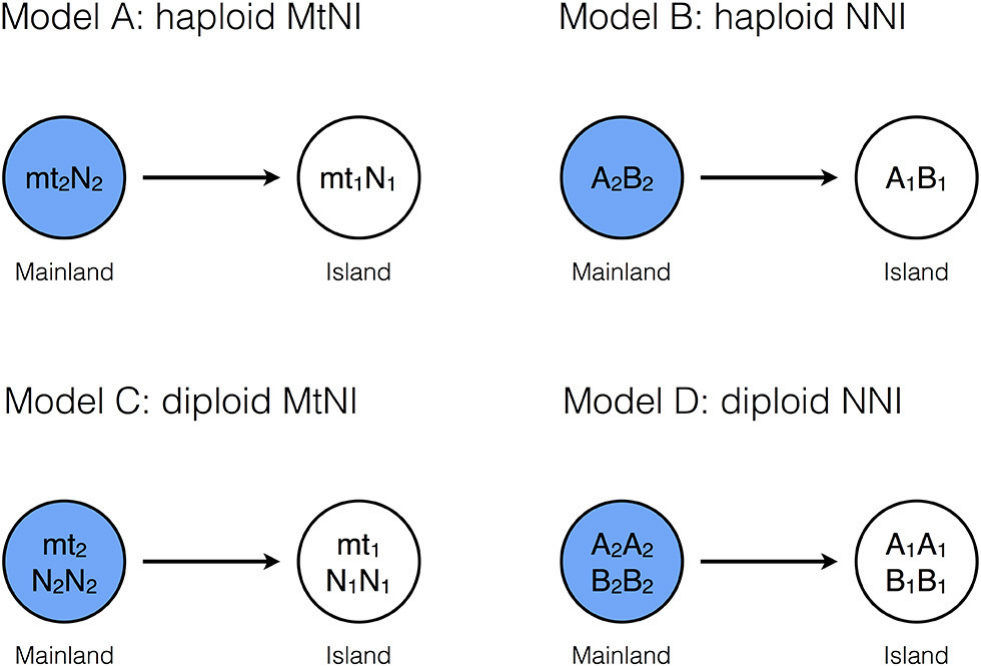
Basic model structures. *Model A:* The island population initially consists only of individuals of genotype mt_1_*N*_1_, whereas the mainland is homogeneous for genotype mt_2_*N*_2_ (haploid genetics). After secondary contact, mating incompatibilities can occur between the mitochondrial haplotype (mt_i_) and the nuclear genotype (*N*_j_). *Model B*: The island population initially consists of individuals of genotype *A*_1_*B*_1_, whereas the mainland is homogeneous for genotype *A*_2_*B*_2_ (haploid genetics). After secondary contact, nuclear-nuclear incompatibilities (NNI) between *A*_i_ and *B*_j_ reduce the number of offspring in intergroup matings. *Model C*: The island population initially consists of individuals of genotype mt_1_*N*_1_*N*_1_, whereas the mainland is homogeneous for genotype mt_2_*N*_2_*N*_2_ (diploid genetics). After secondary, mating incompatibilities can occur between the mitochondrial haplotype (mt_i_) and the nuclear genotype (*N*_j_*N*_k_). *Model D*: The island population initially consists of individuals of genotype *A*_1_*A*_1_*B*_1_*B*_1_, whereas the mainland is homogeneous for genotype *A*_2_*A*_2_*B*_2_*B*_2_ (diploid genetics). After secondary contact, nuclear incompatibilities (NNI) between *A*_i_*A*_j_ and *B*_k_*B*_l_ can occur. For all four models, gene flow was measured by allele frequency changes at a neutrally selected marker locus that is unlinked to the incompatibility loci (Methods). Before secondary contact, the marker allele is at fixation on the mainland, but absent on the island.

We compare four models, which differ in ploidy and/or the mechanisms underlying the incompatibility (Figure 1). The first two assume a haploid-dominant life cycle with a diploid zygote that undergoes meiosis. In *Model A*, incompatibility is caused by mitochondria with two haplotypes mt_1_ and mt_2_, and a nuclear locus with two alleles N_1_ and N_2_ under haploid genetics. The residents in the island population are of type mt_1_N_1_, while the mainland population consists of individuals of type mt_2_N_2_. These pure strains are equally fit and have fitness one. On the other hand, hybrid genotypes mt_1_N_2_ and mt_2_N_1_ suffer from incompatibility and experience reduced relative fitness 1–*s*_1_ and 1–*s*_2_, respectively (Table 1). *Model A* distinguishes between the sexes. Males and females of the same genotype have the same fitness. Differences between the sexes occur because mitochondria are maternally inherited. *Model B* is the same as *Model A* except that two nuclear loci are responsible for the incompatibility and mitochondria play no role. The island and mainland consist of pure strains A_1_B_1_ and A_2_B_2_, and hybrid genotypes A_2_B_1_ and A_1_B_2_ experience reduced relative fitness 1–*s*_A_ and 1–*s*_B_, respectively (Table 2).

**Table 1:** Fitness table for *Model A*.

	***N*_**1**_**	***N*_**2**_**
mt_1_	1	1–*s*_1_
mt_2_	1–*s*_2_	1

**Table 2:** Fitness table for *Model B*.

	***B*_**1**_**	***B*_**2**_**
*A*_1_	1	1–*s*_B_
*A*_2_	1–*s*_A_	1

*Model C* and *Model D* are diploid extensions of *Model A* and *Model B*, respectively. In *Model C*, mitochondria and one nuclear locus are responsible for the reproductive incompatibility. There are two sexes and mitochondria are maternally inherited. The residents in the island population are of type mt_1_N_1_N_1_, while the mainland population consists of type mt_2_N_2_N_2_. Hybrid genotypes undergo reduced fitness according to Table 3, where parameters *h*_1_ and *h*_2_ determine the mode of dominance between the two nuclear loci. In *Model D*, two nuclear loci are responsible for the incompatibility and mitochondria play no role. The residents and migrants have genotypes A_1_A_1_B_1_B_1_ and A_2_A_2_B_2_B_2_, respectively. Fitness reduction in hybrids occurs according to Tables 4, 5, where the parameters *h*_A_ and *h* determine the mode of dominance between the compatible and incompatible nuclear alleles at the same locus. Comparisons between the two models allow us to explore the relative effects of MtNI and NNI on effective migration rates.

**Table 3:** Fitness table for *Model C*.

	***N*_**1**_*N*_**1**_**	***N*_**1**_*N*_**2**_**	***N*_**2**_*N*_**2**_**
mt_1_	1	1–*h*_1_*s*_1_	1–*s*_1_
mt_2_	1–*s*_2_	1–*h*_2_*s*_2_	1

**Table 4:** Fitness table for *Model D*: asymmetric nuclear incompatibilities.

	***B*_**1**_*B*_**1**_**	***B*_**1**_*B*_**2**_**	***B*_**2**_*B*_**2**_**
*A*_1_*A*_1_	1	1	1
*A*_1_*A*_2_	1–*h*_A_*s*_A_	1–*h*_A_*s*_A_	1
*A*_2_*A*_2_	1–*s*_A_	1–*h*_A_*s*_A_	1

**Table 5:** Fitness table for *Model D*: symmetric nuclear incompatibilities.

	***B*_**1**_*B*_**1**_**	***B*_**1**_*B*_**2**_**	***B*_**2**_*B*_**2**_**
*A*_1_*A*_1_	1	1–*hs*	1–*s*
*A*_1_*A*_2_	1–*hs*	1–*hs*	1–*hs*
*A*_2_*A*_2_	1–*s*	1–*hs*	1

In all four models, we have confirmed that the resident island strain is stably maintained against immigration of the mainland strain as long as incompatibility is sufficiently strong and migration is sufficiently low (results not shown). However, the focus of this study is not to investigate the stability of genetic incompatibility in the presence of migration, but to quantify the effect of the incompatibility on gene flow at a neutrally selected nuclear locus, which is not involved in the incompatibility itself. In the next subsections, we explain how we accomplish this goal using the effective migration rate and the fitness graph method.

### Effective Migration Rate

Let us focus on a neutrally selected marker locus unlinked to the incompatibility loci, where we measure gene flow. We denote its frequency in the island population in generation *t* by *p*(*t*) and the frequency of the same allele among immigrants from the mainland population by *q*, where *q* is assumed to be constant. The effective migration rate with respect to the focal marker locus is then defined as (Kobayashi et al., 2008):

(1) $$\begin{array}{r} m_{e}=\underset{t\to \infty }{\text{lim}}\frac{p\left(t+1\right)-p\left(t\right)}{q-p\left(t\right)} \end{array}$$

The numerator on the right hand side of Equation (1) is the per generation increase in allele frequency of the marker allele on the island. Note that this term is equal to the per generation decrease in the difference in allele frequency between residents and migrants, i.e., (*q*–*p*(*t*))–(*q*–*p*(*t*+1)). Note further that the denominator in Equation (1) is the difference in allele frequency between residents and migrants. Thus, the effective migration rate measures the gene flow by the relative rate at which the frequency difference between residents and migrants decreases.

It can be shown under quite general conditions that the effective migration rate *m*_*e*_ agrees with the actual migration rate *m* if there is no gene flow modification (Kobayashi et al., 2008). For models with gene flow modification, the ratio *m*_*e*_*/m* gives a normalized effect of the gene flow modifier and is called the *gene flow factor* (Bengtsson, 1985). Gene flow reduction is indicated by gene flow factors smaller than one, and gene flow enhancement by gene flow factors larger than one.

The effective migration rate can be calculate numerically by explicitly writing down recursion equations and tracking the allele frequencies of the marker allele through time by computer simulations. We have done such an analysis for specific parameter constellations of all four models. However, these numerical results are only shown in the supplement because large-scale parameters screenings were necessary to make general conclusions. Instead, we conducted an analytical analysis using the fitness graph method (see section Fitness Graph Method), and used the numerical analysis only to test the accuracy of the derived formulae. For all four models investigated, we have checked that our analytical formulae fit well with the numerical results obtained from the recursion equations, i.e., the numerical estimates of the effective migration rate converge to the analytical formulae if the migration rate converges to zero (Supplementary Material S1, Figure S4).

### Fitness Graph Method

In a previous study (Kobayashi et al., 2008), we derived the following general approximation formula for the effective migration rate *m*_*e*_, which works well for a broad range of mainland-island models as long as the migration rate *m* is small. It holds that

(2) $$\begin{array}{r} \underset{m\to 0}{\lim}\frac{m_{e}}{m}=\nu , \end{array}$$

where *v* is the average reproductive value of migrants. Within this framework, the reproductive values are normalized relative to residents, implying that the reproductive value of residents is equal to one.

Equation (2) connects the effective migration rate to the concept of reproductive value. In general, the reproductive value of an individual gives the long-term genetic contribution of the individual to the gene pool of the population (Fisher, 1930; Caswell, 1982; Taylor, 1990). Equation (2) shows that gene flow (as quantified by the effective migration rate) is well-approximated by the product of the migration rate and the reproductive value of migrants if migration is weak. Therefore, the problem of calculating the effective migration rate reduces to the problem of calculating reproductive values of migrants. For this purpose several approaches are available, which are all mathematically identical (Kobayashi and Telschow, 2008). In this article we use the so-called fitness-graph method (Kobayashi and Telschow, 2008). A hidden assumption of the method is that migration is so low that migrants and their entire future progenies mate solely with residents.

The fitness-graph method allows computing the reproductive values of individuals in different classes of a population using a graph associated with the specific model in question. Computing reproductive values is essentially an eigenvalue problem (Caswell, 1982; Taylor, 1990, 1996; Rousset, 2004; Kobayashi and Telschow, 2008; Kobayashi et al., 2008), and the fitness graph method solves this problem in an intuitive manner by expressing the matrix involved in the problem as a directed graph. From the viewpoint of alleles at the neutral marker locus, different genotypes at incompatibility loci can be viewed as different backgrounds or classes between which the alleles can move, just like different sexes or age classes (see section Results in Kobayashi et al., 2008, where sexes and genotypes are both treated as classes in the same framework). Therefore, we can define the reproductive value for each genotype at the incompatibility loci as the average long-term contribution of individuals of the genotype to the gene pool of the marker locus and compute it using the fitness-graph method (Kobayashi et al., 2008).

The fitness graph analysis consists of five steps (Kobayashi and Telschow, 2008). First, the different genetic classes of the model are written down. Second, a fitness graph is drawn that describes how the different genetic classes relate to each other. In the fitness graph, arrows are drawn from parent to offspring genotypes under the assumption that mating occurs solely and at random with resident individuals. The numbers attached to the arrows correspond to the fitness of the offspring genotypes. Third, the fitness graph is translated into a system of coupled linear equations. The number of equations equals the number of genetic classes, and the unknown variables are the reproductive values of the different genetic classes. Fourth, analytical formula for the reproductive values are derived by solving the equation system. Fifth, the reproductive values of the genotype classes are used to determine the average reproductive value of migrants.

## Results

For all four models, we quantified gene flow from the mainland to the island by calculating the average reproductive value of migrants (*v*). We allowed for sex ratio biases among migrants. The parameter *m*_*f*_ describes the fraction of females among migrants. The average reproductive value *v* was calculated using the fitness graph method, and under the assumption that migration is so low that migrants and their entire future progenies mate solely with residents.

### Models With Haploid Genetics

#### Model A: Mito-Nuclear Incompatibilities Under Haploid Genetics

##### Analytical formulae for gene flow reduction

We first analyzed the effect of mito-nuclear incompatibilities (MtNI) on gene flow in a mainland-island model with haploid genetics (Figure 1, *Model A*). Eight genetic classes need to be considered because there are four different genotypes and males and females have different fitness. The reproductive values are denoted by $v_{mt_{i}N_{j}}^{\text{♂}}$ and $v_{mt_{i}N_{j}}^{\text{♀}}$, where *i* indicates the mitochondrial haplotype, *j* the nucleotype, and ♂ and ♀ the male and female sex, respectively. As explained above, we assume that the reproductive values of island residents are equal to one, i.e., $v_{mt_{1}N_{1}}^{\text{♂}}=v_{mt_{1}N_{1}}^{\text{♀}}=\;1$.

**Figure 2:**
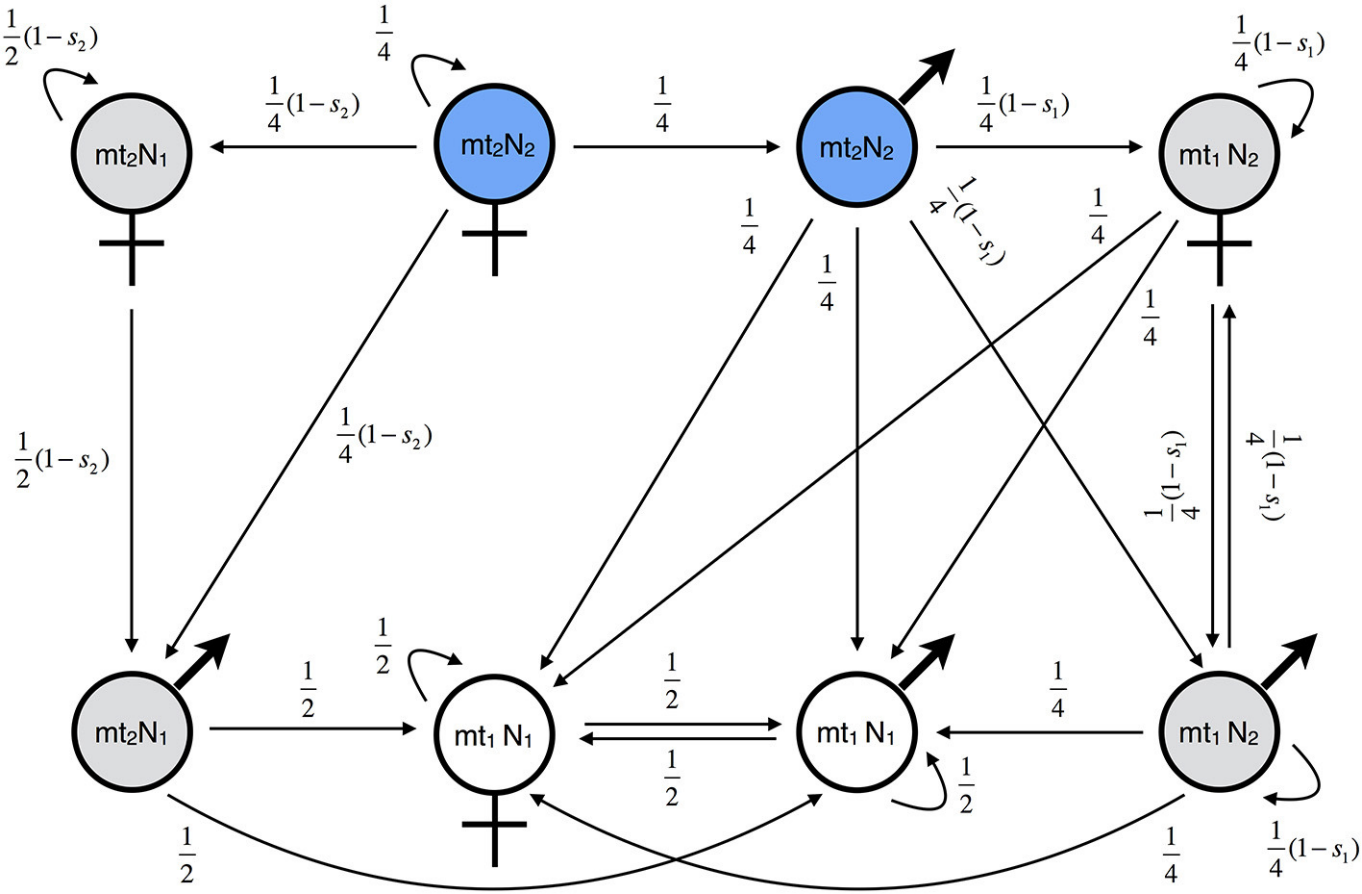
Fitness graph for haploid MtNI. Shown is the fitness graph for *Model A* (mito-nuclear incompatibilities, haploid genetics). There are eight genetic classes. Class transitions are indicated by black arrows and attached weighting factors. The edges correspond to the proportion of offspring after a cross with a resident individual, weighted by the fitness of the resulting genotype. The graph describes gene flow of rare male and female migrants of genotype mt_2_N_2_ in an island population consisting of mostly mt_1_N_1_ individuals. Blue indicates the migrant genotypes, gray the hybrid genotypes, and white the resident genotypes. Gene flow is measured at a neutrally selected nuclear marker locus, which is not shown in the graph (Methods).

The reproductive values of the other classes were calculated analytically using the fitness graph shown in Figure 2. The graph describes how the eight genetic classes relate to each other. The arrows represent parent-offspring relationships between eight genotype classes. The number attached to the arrow from genotype *X* to *Y* represents the fitness component of a genotype-*X* parent attributed to genotype-*Y* offspring, where fitness components are normalized such that the fitness of a resident is one. Precisely speaking, the fitness component here is the relative number of *adult offspring* of genotype *Y*, which is given by the fraction of genotype-*Y* offspring among all the offspring of the genotype-*X* parent multiplied by the viability of offspring genotype *Y*. For example, in the figure, the arrow from the *mt*_2_*N*_2_ female to the *mt*_2_*N*_1_ male comes with fitness component (1/2)(1–*s*_2_) because the half of the offspring of an *mt*_2_*N*_2_ female are *mt*_2_*N*_1_ males, given that she mates exclusively with *mt*_1_*N*_1_ males, and the viability of those male offspring is 1–*s*_2_. The graph can be translated into a system of linear equations, noting that the genetic contribution of an individual to the gene pool of the marker locus must be equal to the total genetic contribution of his/her offspring to the gene pool. In other words, the reproductive value of genotype *X* must be equal to the weighted sum of the reproductive values of all the genotypes pointed by an arrow from *X*, where the weights are the corresponding fitness components. Applying this principle to Figure 2 yields

(3) $$\begin{array}{r} v_{mt_{1}N_{1}}^{\text{♂}}=1 \end{array}$$

(4) $$\begin{array}{r} v_{mt_{1}N_{1}}^{\text{♀}}=1, \end{array}$$

(5) $$\begin{array}{l} v_{mt_{1}N_{2}}^{\text{♂}}=\frac{1}{4}v_{mt_{1}N_{1}}^{\text{♂}}+\frac{1}{4}\left(1-s_{1}\right)v_{mt_{1}N_{2}}^{\text{♂}}+\frac{1}{4}v_{mt_{1}N_{1}}^{\text{♀}}\\ \;+\frac{1}{4}\left(1-s_{1}\right)v_{mt_{1}N_{2}}^{\text{♀}}, \end{array}$$

(6) $$\begin{array}{l} v_{mt_{1}N_{2}}^{\text{♀}}=\frac{1}{4}v_{mt_{1}N_{1}}^{\text{♂}}+\frac{1}{4}\left(1-s_{1}\right)v_{mt_{1}N_{2}}^{\text{♂}}+\frac{1}{4}v_{mt_{1}N_{1}}^{\text{♀}}\\ \;+\frac{1}{4}\left(1-s_{1}\right)v_{mt_{1}N_{2}}^{\text{♀}}, \end{array}$$

(7) $$\begin{array}{l} v_{mt_{2}N_{1}}^{\text{♂}}=\frac{1}{2}v_{mt_{1}N_{1}}^{\text{♂}}+\frac{1}{2}v_{mt_{1}N_{1}}^{\text{♀}}, \end{array}$$

(8) $$\begin{array}{l} v_{mt_{2}N_{1}}^{\text{♀}}=\frac{1}{2}\left(1-s_{2}\right)v_{mt_{2}N_{1}}^{\text{♂}}+\frac{1}{2}\left(1-s_{2}\right)v_{mt_{2}N_{1}}^{\text{♀}}, \end{array}$$

(9) $$\begin{array}{l} v_{mt_{2}N_{2}}^{\text{♂}}=\frac{1}{4}v_{mt_{1}N_{1}}^{\text{♂}}+\frac{1}{4}\left(1-s_{1}\right)v_{mt_{1}N_{2}}^{\text{♂}}+\frac{1}{4}v_{mt_{1}N_{1}}^{\text{♀}}\\ \;+\frac{1}{4}\left(1-s_{1}\right)v_{mt_{1}N_{2}}^{\text{♀}}, \end{array}$$

(10) $$\begin{array}{l} v_{mt_{2}N_{2}}^{\text{♀}}=\frac{1}{4}\left(1-s_{2}\right)v_{mt_{2}N_{1}}^{\text{♂}}+\frac{1}{4}v_{mt_{2}N_{2}}^{\text{♂}}+\frac{1}{4}{\left(1-s_{2}\right)v}_{mt_{2}N_{1}}^{\text{♀}}\\ \;+\frac{1}{4}v_{mt_{2}N_{2}}^{\text{♀}}. \end{array}$$

There is one unique solution to the equation system (3–10). Straightforward algebraic calculations reveal analytical formulae of the reproductive values of the male and female migrants. These compute to

(11) $$\begin{array}{l} v_{mt_{2}N_{2}}^{\text{♂}}=\frac{1}{1+s_{1}}, \end{array}$$

(12) $$\begin{array}{l} v_{mt_{2}N_{2}}^{\text{♀}}=\frac{3+{2s}_{1}-s_{2}-2s_{1}s_{2}}{3\left(1+s_{1}\right)\left(1+s_{2}\right)}. \end{array}$$

Formulae (11, 12) show substantial differences between male and female migrants. The male reproductive value depends only on parameter *s*_1_, but the female reproductive values on both *s*_1_ and *s*_2_. This asymmetry is also visible in the fitness graph (Figure 2). The class of male migrants has four links, whereas the class of female migrants has only three. This is in stark contrast to nuclear-nuclear incompatibilities, for which the fitness graph is symmetric (Figure S1).

From Equations (11, 12), we get analytical formulae of the average reproductive value of migrants. These compute to

(13) $$\begin{array}{l} v=\left(1-m_{f}\right)v_{mt_{2}N_{2}}^{\text{♂}}+m_{f}v_{mt_{2}N_{2}}^{\text{♀}}, \end{array}$$

(14) $$\begin{array}{l} v=\left(1-m_{f}\right)\frac{1}{1+s_{1}}+m_{f}\frac{3+{2s}_{1}-s_{2}-2s_{1}s_{2}}{3\left(1+s_{1}\right)\left(1+s_{2}\right)}. \end{array}$$

For the analysis below, we pay special attention to three cases: (i) symmetric MtNI, i.e., *s* = *s*_1_ = *s*_2_, (ii) asymmetric incompatibility of type I, i.e., *s*_1_ = 0, *s*_2_ > 0, and (iii) asymmetric incompatibility of type II, *s*_1_ > 0, *s*_2_ = 0. For these cases equation (11) and (12) reduce to $v_{mt_{2}N_{2}}^{\text{♂}}=\frac{1}{1+s}$ and $v_{mt_{2}N_{2}}^{\text{♀}}=\frac{3-2s}{3\left(1+s\right)}$ for symmetric MtNI, $v_{mt_{2}N_{2}}^{\text{♂}}=1$ and $v_{mt_{2}N_{2}}^{\text{♀}}=\frac{3-s_{2}}{3\left(1+s_{2}\right)}$ for asymmetric MtNI of type I, and $v_{mt_{2}N_{2}}^{\text{♂}}=\frac{1}{1+s_{1}}$ and $v_{mt_{2}N_{2}}^{\text{♀}}=\frac{3+{2s}_{1}}{3\left(1+s_{1}\right)}$ for asymmetric MtNI of type II.

##### Quantitative analysis of gene flow reduction

**Figure 3:**
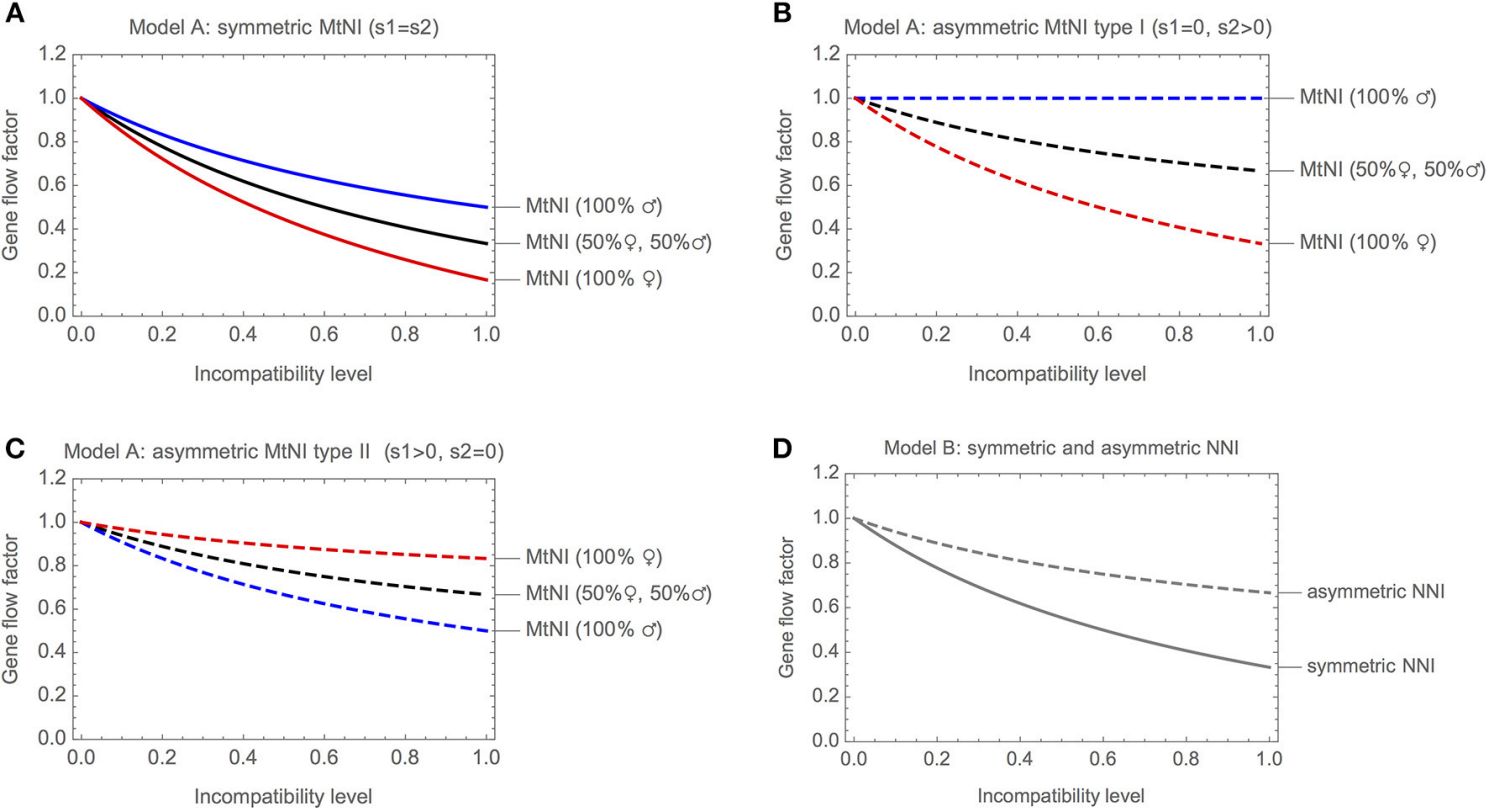
Gene flow reduction for models with haploid genetics. Shown is the gene flow factor (values below 1 indicate a reduction of gene flow) for an unlinked neutral locus as a function of the level of incompatibility. **(A)** *Model A* with symmetric MtNI and varying incompatibility level *s*_1_ = *s*_2_. **(B)** *Model A* with asymmetric MtNI of type I (*s*_1_ = 0) and varying incompatibility level *s*_2_. **(C)** *Model A* with asymmetric MtNI of type II (*s*_2_ = 0) and varying incompatibility level *s*_1_. **(D)** *Model B* with symmetric (*s*_A_ = *s*_B_) and asymmetric (*s*_B_ = 0) NNI. For the former, the gene flow factor is shown as a function of *s*_A_ = *s*_B_, for the latter as a function of *s*_A_.

Figure 3A shows the gene flow factor (average reproductive value of migrants) as a function of the level of incompatibility for symmetric MtNI. Shown are three scenarios: (i) only females migrate, (ii) only males migrate, and (iii) males and females migrate in equal number. For all three scenarios, we found that gene flow at the unlinked nuclear marker locus is a monotonously decreasing function of the level of incompatibility. However, gene flow is most strongly reduced if only females migrate and least if only males migrate. The maximal reduction in gene flow at the unlinked nuclear marker locus is 83.3% if only females migrate, 50% if only males migrate, and 66.6% if males and females migrate in equal number. The basic reason for this sex difference is the maternal inheritance of mitochondria. It results in strong coupling of the migrant's marker allele and the mitochondrial haplotype mt_2_ in the female line. In contrast, the association between the marker allele and mt_2_ is already lost in the F1 generation of male migrants. This effect is visible in the fitness graph (Figure 2).

Figure 3B shows the gene flow factor for the same three scenarios, but now for asymmetric MtNI of type I, where mt_2_ is incompatible with N_1_ but mt_1_ is fully compatible with N_2_. We found that gene flow is most strongly reduced if only females migrate and least if only males migrate. This is qualitatively similar to symmetric MtNI. However, gene flow reduction is generally lower than in Figure 3A with a maximal reduction in gene flow of 66.7% if only females migrate and 33.3% if males and females migrate in equal number. Remarkably, there is no gene flow reduction at the unlinked neutral nuclear locus if only males migrate. The reason for this is that the locus becomes uncoupled with the mitochondrial incompatibility haplotype (mt_2_) in the first hybrid generation following migration (cf. Figure 2).

Figure 3C shows the gene flow factor for asymmetric MtNI of type II, where mt_1_ is incompatible with N_2_ but mt_2_ is fully compatible with N_1_. Here, we found that gene flow at the unlinked neutral nuclear locus is most strongly reduced if only males migrate and least if only females migrate. This is the opposite of what we found for symmetric MtNI and asymmetric MtNI of type I. The maximal reduction in gene flow for type II is 16.7% if only females migrate, 50% if only males migrate, and 33.3% if males and females migrate in equal number. Thus, there are substantial differences between the two types of asymmetric MtNI. Nevertheless, we found that gene flow reduction is exactly the same for both types if males and females migrate in equal number.

#### Model B: Nuclear-Nuclear Incompatibilities Under Haploid Genetics

##### Analytical formulae for gene flow reduction

We then conducted the same analysis for a scenario, in which gene flow reduction occurs due to nuclear-nuclear incompatibilities (*Model B*, Figure 1). In this model, there are only four genetic classes. In contrast to *Model A*, males and females do not need to be distinguished because they have the same fitness. The reproductive values of the four genetic classes are denoted by *v*_*A*_1_*B*_1__, *v*_*A*_1_*B*_2__, *v*_*A*_2_*B*_1__, and *v*_*A*_2_*B*_2__. As above, we assume that the reproductive value of the resident genotype (*A*_1_*B*_1_) is equal to one. The other reproductive values are calculated analytically using the fitness graph shown in Figure S1. From the fitness graph, we derived a system of four linear equations that describe the transition between the four genetic classes. These compute to

(15) $$\begin{array}{l} v_{A_{1}B_{1}}=1, \end{array}$$

(16) $$\begin{array}{l} v_{A_{1}B_{2}}=\frac{1}{2}\left(1-s_{B}\right)v_{A_{1}B_{2}}+\frac{1}{2}v_{A_{1}B_{1}}, \end{array}$$

(17) $$\begin{array}{l} v_{A_{2}B_{1}}=\frac{1}{2}\left(1-s_{A}\right)v_{A_{2}B_{1}}+\frac{1}{2}v_{A_{1}B_{1}}, \end{array}$$

(18) $$\begin{array}{l} v_{A_{2}B_{2}}=\frac{1}{4}v_{A_{2}B_{2}}+\frac{1}{4}\left(1-s_{A}\right)v_{A_{2}B_{1}}\\ \;+\frac{1}{4}\left(1-s_{B}\right)v_{A_{1}B_{2}}+\frac{1}{4}v_{A_{1}B_{1}}. \end{array}$$

This system of linear equations has a unique solution. Straightforward algebraic calculations reveal that

(19) $$\begin{array}{l} v_{A_{1}B_{2}}=\frac{1}{1+s_{B}}, \end{array}$$

(20) $$\begin{array}{l} v_{A_{2}B_{1}}=\frac{1}{1+s_{A}}, \end{array}$$

(21) $$\begin{array}{l} v_{A_{2}B_{2}}=\frac{3+s_{A}+s_{B}-s_{A}s_{B}}{3\left(1+s_{A}+s_{B}+s_{A}s_{B}\right)}. \end{array}$$

For comparison with *Model A*, we pay special attention to symmetric (i.e., *s* = *s*_A_ = *s*_B_) and asymmetric incompatibilities (i.e., *s*_A_ > 0, *s*_B_ = 0). Equation (21) reduces to $v_{A_{2}B_{2}}=\frac{3-s}{3+3s}$ for symmetric NI and $v_{A_{2}B_{2}}=\frac{3+s_{A}}{3+3s_{A}}$ for asymmetric NI.

##### Quantitative analysis of gene flow reduction

Figure 3D shows the gene flow factor as a function of the level of incompatibility. As in *Model A*, gene flow is a monotonously decreasing function of the level of incompatibility. For symmetric NNI, gene flow is maximally reduced by 66.7%. In this case, it holds that $v_{A_{2}B_{2}}=\frac{1}{3}$. For asymmetric NNI, gene flow is maximally reduced by 33.3%. In this case, it holds that $v_{A_{2}B_{2}}=\frac{2}{3}$.

#### Comparison of Model A and B

We compared the results of *Model B* with that of *Model A*. Interestingly, gene flow reduction is exactly the same for NNI and MtNI as long as males and females migrate in equal number. This follows from the fact that the right hand sides of Equation (21) and Equation (14) are equal if *s*_1_ = *s*_A_, *s*_2_ = *s*_B_, and *m*_*f*_ = 0.5. However, when the sex ratio among migrants is biased, differences occur between the models. For most parameter constellations, we found that gene flow reduction at an unlinked neutral locus is stronger or (weaker) for MtNI than for NNI if more (less) females migrate than males. This finding reflects the maternal inheritance of mitochondria and is a fundamental difference between MtNI and NNI.

### Models With Diploid Genetics

*Models A* and *B* follow haploid genetics. In order to test whether the main conclusions drawn from these models depend on the ploidy of the system, we further analyzed their diploid extensions (Figure 1, *Model C* and *D*). These diploid models allowed further investigating how dominance affects the results.

#### Model C: Mito-Nuclear Incompatibilities Under Diploid Genetics

##### Analytical formulae for gene flow reduction

For *Model C*, we conducted the same basic analysis as above. Using a fitness graph (Figure S2), we derived a system of ten linear equations that describes how the reproductive values of the different genetic classes relate to each other. It holds that

(22) $$\begin{array}{l} v_{mt_{1}N_{1}N_{1}}^{\text{♂}}=1, \end{array}$$

(23) $$\begin{array}{l} v_{mt_{1}{N_{1}N}_{1}}^{\text{♀}}=1, \end{array}$$

(24) $$\begin{array}{l} v_{mt_{1}N_{1}N_{2}}^{\text{♂}}=\frac{1}{2}+\frac{1}{4}\left(1-h_{1}s_{1}\right)v_{mt_{1}N_{1}N_{2}}^{\text{♂}}+\frac{1}{4}\left(1-h_{1}s_{1}\right)v_{mt_{1}N_{1}N_{2}}^{\text{♀}} \end{array}$$

(25) $$\begin{array}{l} v_{mt_{1}N_{1}N_{2}}^{♀}=\frac{1}{2}+\frac{1}{4}(1-h_{1}s_{1})v_{mt_{1}N_{1}N_{2}}^{♂}\\ \;+\frac{1}{4}(1-h_{1}s_{1})v^{♀}mt_{1}N_{1}N_{2},v_{mt_{2}N_{1}N_{1}}^{♂}=1, \end{array}$$

(26) $$\begin{array}{l} v_{mt_{2}N_{1}N_{1}}^{\text{♀}}=\frac{1}{2}\left(1-s_{2}\right)v_{mt_{2}N_{1}N_{1}}^{\text{♂}}+\frac{1}{2}\left(1-s_{2}\right)v_{mt_{2}N_{1}N_{1}}^{\text{♀}}, \end{array}$$

(27) $$\begin{array}{l} v_{mt_{2}N_{1}N_{2}}^{\text{♂}}=\frac{1}{2}+\frac{1}{4}\left(1-h_{1}s_{1}\right)v_{mt_{1}N_{1}N_{2}}^{\text{♂}}+\frac{1}{4}\left(1-h_{1}s_{1}\right)v_{mt_{1}N_{1}N_{2}}^{\text{♀}}, \end{array}$$

(28) $$\begin{array}{l} v_{mt_{2}N_{1}N_{2}}^{\text{♀}}=\frac{1}{4}\left(1-h_{2}s_{2}\right)v_{mt_{2}N_{1}N_{2}}^{\text{♂}}+\frac{1}{4}\left(1-h_{2}s_{2}\right)v_{mt_{2}N_{1}N_{2}}^{\text{♀}}\\ \;+\frac{1}{4}\left(1-s_{2}\right)v_{mt_{2}N_{1}N_{1}}^{\text{♂}}+\frac{1}{4}\left(1-s_{2}\right)v_{mt_{2}N_{1}N_{1}}^{\text{♀}}, \end{array}$$

(29) $$\begin{array}{l} v_{mt_{2}N_{2}N_{2}}^{\text{♂}}=\frac{1}{2}\left(1-h_{1}s_{1}\right)v_{mt_{1}N_{1}N_{2}}^{\text{♂}}+\frac{1}{2}\left(1-h_{1}s_{1}\right)v_{mt_{1}N_{1}N_{2}}^{\text{♀}}, \end{array}$$

(30) $$\begin{array}{l} v_{mt_{2}N_{2}N_{2}}^{\text{♀}}=\frac{1}{2}\left(1-h_{2}s_{2}\right)v_{mt_{2}N_{1}N_{2}}^{\text{♂}}+\frac{1}{2}\left(1-h_{2}s_{2}\right)v_{mt_{2}N_{1}N_{2}}^{\text{♀}}. \end{array}$$

There is one unique solution to the equation system (22–30). Straightforward algebraic calculations reveal analytical formulae of the reproductive values of the male and female migrants. These compute to

(31) $$\begin{array}{l} v_{mt_{2}N_{2}N_{2}}^{\text{♂}}=\frac{1-h_{1}s_{1}}{1+{h_{1}s}_{1}}, \end{array}$$

(32) $$\begin{array}{l} v_{mt_{2}N_{2}N_{2}}^{\text{♀}}=\frac{\left(1-h_{2}s_{2}\right)\left(3+s_{2}+h_{1}s_{1}\left(1-s_{2}\right)\right)}{\left(1+h_{1}s_{1}\right)\left(1+s_{2}\right)\left(3+h_{2}s_{2}\right)}. \end{array}$$

Formulas (31, 32) show substantial differences between male and female migrants. As for *Model A*, the male reproductive value depends only on parameter *s*_1_, but the female reproductive value on both *s*_1_ and *s*_2_. This asymmetry is also visible in the fitness graph (Figure S2). Note that the reproductive value of male migrants is the same as for diploid NNI (see below).

From Equations (31, 32), we get analytical formulae of the average reproductive value of migrants. These compute to

(33) $$\begin{array}{l} v=\left(1-m_{f}\right)v_{mt_{2}N_{2}N_{2}}^{\text{♂}}+m_{f}v_{mt_{2}N_{2}N_{2}}^{\text{♀}}, \end{array}$$

(34) $$\begin{array}{l} v=\left(1-m_{f}\right)\frac{1-h_{1}s_{1}}{1+{h_{1}s}_{1}}+m_{f}\frac{\left(1-h_{2}s_{2}\right)\left(3+s_{2}+h_{1}s_{1}\left(1-s_{2}\right)\right)}{\left(1+h_{1}s_{1}\right)\left(1+s_{2}\right)\left(3+h_{2}s_{2}\right)}. \end{array}$$

For the analysis below, we pay special attention to two cases: (i) recessive MtNI, i.e. *h*_1_ = *h*_2_ = 0 and (ii) dominant MtNI, i.e., *h*_1_ = *h*_2_ = 1. For these cases equation (30) and (31) reduce to $v_{mt_{2}{N_{2}N}_{2}}^{\text{♂}}=1$ and $v_{mt_{2}N_{2}N_{2}}^{\text{♀}}=\frac{3+s_{2}}{3+3s_{2}}$ for recessive MtNI, and $v_{mt_{2}N_{2}N_{2}}^{\text{♂}}=\frac{1-s_{1}}{1+s_{1}}$ and $v_{mt_{2}{N_{2}N}_{2}}^{\text{♀}}=\frac{\left(1-s_{2}\right)\left(3+s_{2}+s_{1}\left(1-s_{2}\right)\right)}{\left(1+s_{1}\right)\left(1+s_{2}\right)\left(3+s_{2}\right)}$ for dominant MtNI.

##### Quantitative analysis of gene flow reduction

**Figure 4:**
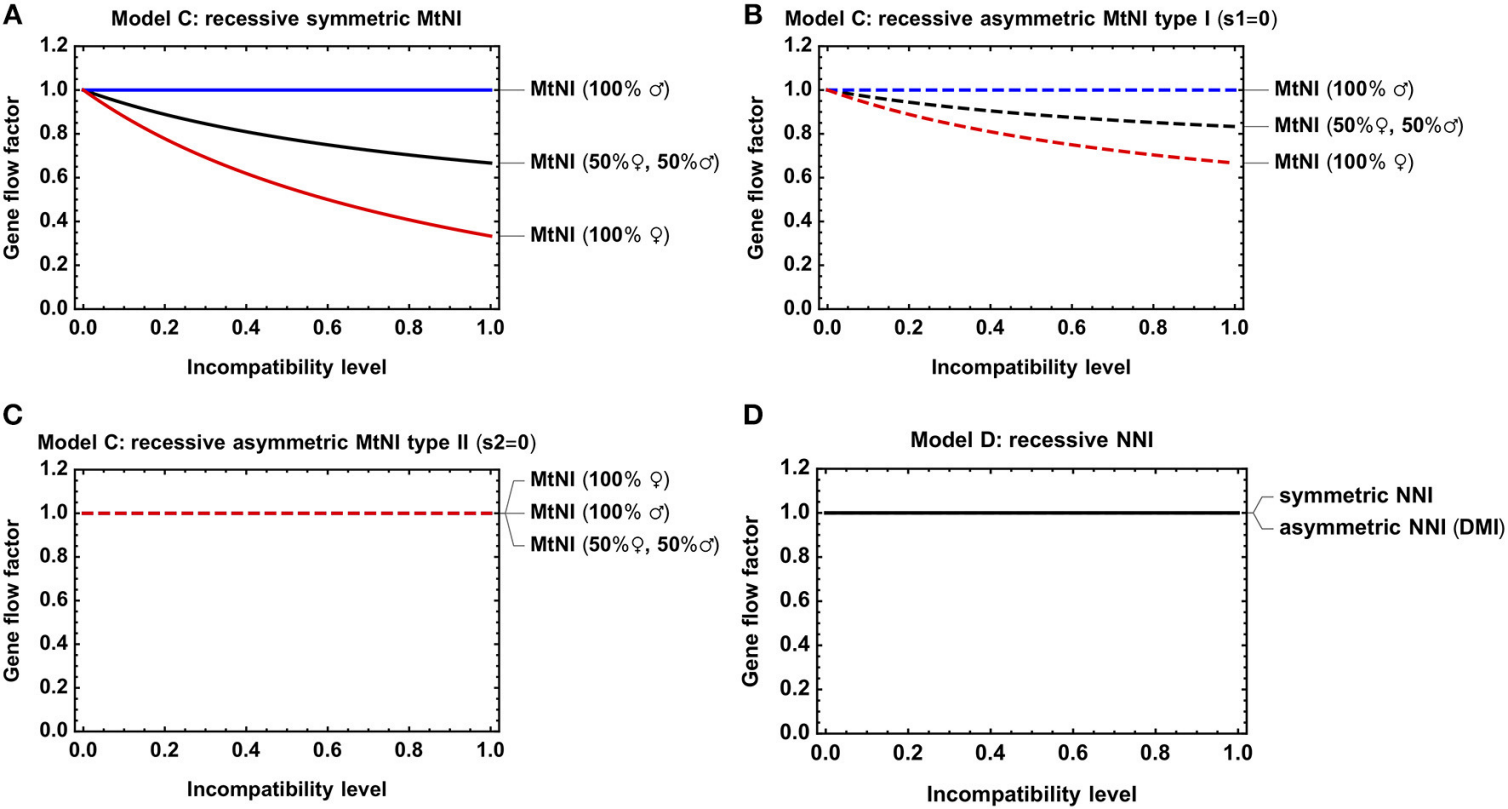
Gene flow reduction for models with diploid genetics. Shown is the gene flow factor for an unlinked neutral locus as a function of the level of incompatibility. **(A)** *Model C* with recessive symmetric MtNI and varying incompatibility level *s*_1_ = *s*_2_. **(B)** *Model C* with recessive asymmetric MtNI of type I (*s*_1_ = 0) and varying incompatibility level *s*_2_. **(C)** *Model C* with recessive asymmetric MtNI of type II (*s*_2_ = 0) and varying incompatibility level *s*_1_. **(D)** *Model D* with symmetric (*s*_A_ = *s*_B_) and asymmetric (*s*_B_ = 0) NNI. For the former, the gene flow factor is shown as a function of *s*_A_ = *s*_B_, for the latter as a function of *s*_A_. Parameters: *h* = *h*_*A*_ = 0.

A main conclusion from *Model A* was that the sex ratio among migrants significantly affects the results (Figures 3A–C). Our analysis of *Model C* revealed the same basic trends, irrespective of whether mito-nuclear incompatibilities are recessive (Figures 4A–C) or dominant (Figures S3A–C). As in *Model A* and for the same reasons discussed there, we found (i) that symmetric MtNI and asymmetric MtNI of type I result in stronger gene flow reduction when more females migrate than males, and weaker in the reciprocal case of more male migration (Figures 4A,B, Figures S3A,B), and that (ii) the opposite pattern occurs for asymmetric MtNI of type II (Figure 4C, Figure S3C).

**Figure 5:**
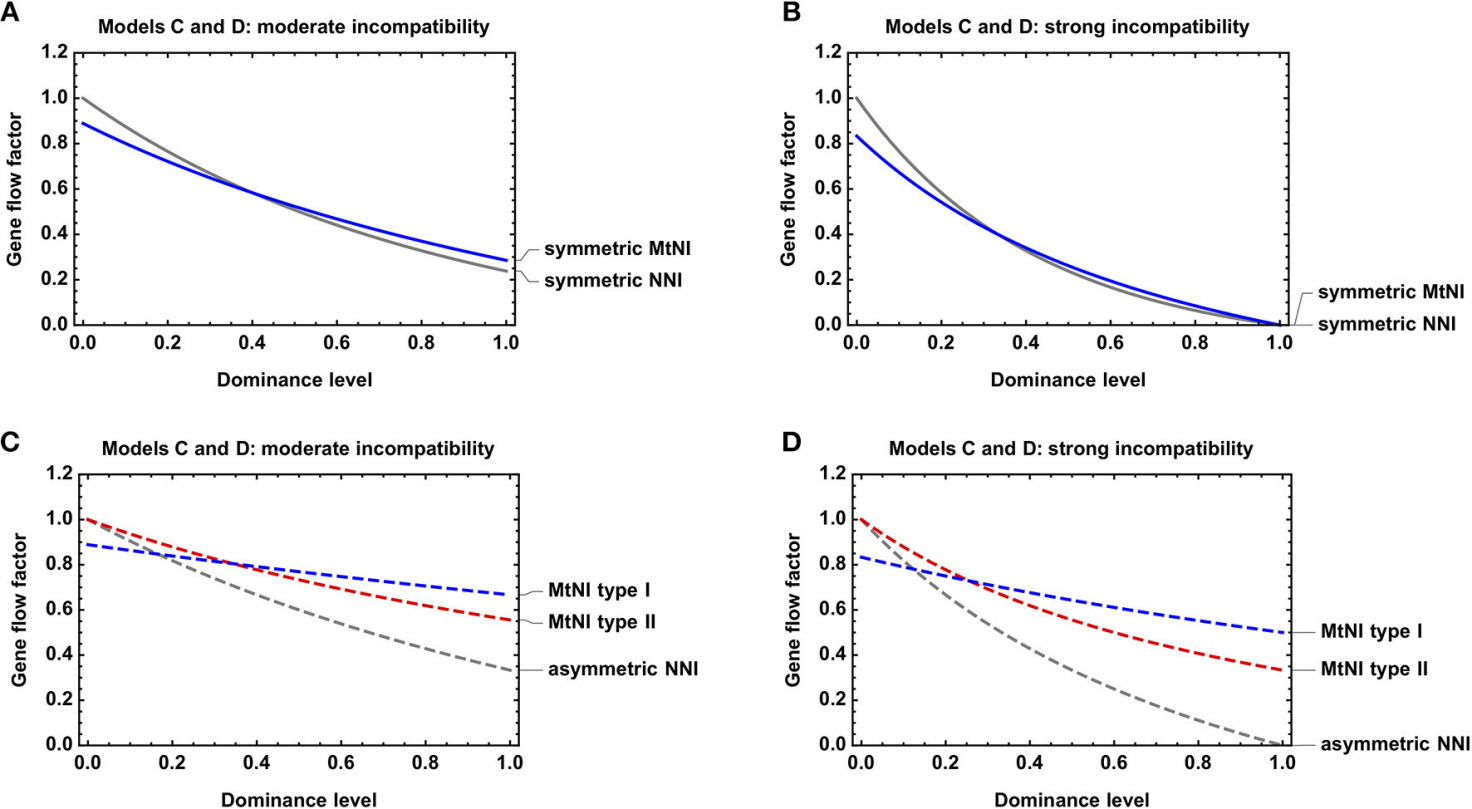
Effect of dominance on gene flow reduction. Shown is the gene flow factor as a function of the dominance levels for *Models C* and *D*. **(A)** Symmetric MtNI vs. symmetric NNI for *s* = *s*_1_ = *s*_2_ = *s*_*A*_ = 0.5. **(B)** Symmetric MtNI vs. symmetric NNI for *s* = *s*_1_ = *s*_2_ = *s*_*A*_ = 1. **(C)** Asymmetric MtNI vs. asymmetric NNI for *s* = *s*_1_ = *s*_2_ = *s*_*A*_ = 0.5. **(D)** Asymmetric MtNI vs. asymmetric NNI for *s* = *s*_1_ = *s*_2_ = *s*_*A*_ = 1. The number of male and female migrants is equal for *Model C*, i.e., *m*_*f*_ = 0.5.

We further investigated the effect of dominance on the strength of gene flow reduction. As expected, gene flow reduction is lowest for recessive MtNI and increases monotonously with the level of dominance (Figure 5).

#### Model D: Nuclear-Nuclear Incompatibilities Under Diploid Genetics

*Model D* was previously analyzed with respect to gene flow reduction (Gavrilets, 1997; Kobayashi and Telschow, 2008). Let *v*_*A*_2_*A*_2_*B*_2_*B*_2__ denote the reproductive value of the migrants. Then it holds that

(35) $$\begin{array}{l} v_{A_{2}A_{2}B_{2}B_{2}}=\frac{\left(1-h_{A}s\right)\left(3+2h_{A}s-h_{A}^{2}s^{2}\right)}{{\left(1+h_{A}s_{A}\right)}^{2}\left(3+h_{A}s\right)} \end{array}$$

for asymmetric NNI (Table 4), and

(36) $$\begin{array}{l} v_{A_{2}A_{2}B_{2}B_{2}}=\frac{1-hs_{A}}{1+hs_{A}} \end{array}$$

for symmetric NNI (Table 5). Note that *Model D* with asymmetric NNI is a special case of the Dobzhansky-Muller model.

Although *Model D* contains in total nine genetic classes, the fitness graph of the A_2_A_2_B_2_B_2_ migrants contains only seven. These are A_1_A_1_B_1_B_1_, A_1_A_1_B_1_B_2_, A_1_A_2_B_1_B_1_, A_1_A_2_B_1_B_2_, A_1_A_2_B_2_B_2_, A_2_A_2_B_1_B_2_, and A_2_A_2_B_2_B_2_. The genetic classes A_1_A_1_B_2_B_2_ and A_2_A_2_B_1_B_1_ do not belong to the fitness graph because migrants of genotype A_2_A_2_B_2_B_2_ do not have progenies of these genotypes. The reason is that all migrants and their offspring are assumed to be sufficiently rare in the population that they mate solely with residents of genotype A_1_A_1_B_1_B_1_. There is one important implication of this assumption. Recessive NNI does not result in gene flow reduction because, under recessive NNI, only genotypes A_1_A_1_B_2_B_2_ and A_2_A_2_B_1_B_1_ have reduced fertility, but these genotypes can never be progenies of the A_2_A_2_B_2_B_2_ migrants (Figure 4D). Thus, the model does not address the circumstances where migration is sufficiently strong that these recessive genotypes become substantial in the population.

#### Comparison of Model C and D

Finally, we compared *Model C* and *D* with respect to dominance. For both models, we found that gene flow reduction is monotonously increasing with the level of dominance. This is true for both symmetric and asymmetric incompatibilities (Figure 5). However, large quantitative differences occur between the models with respect to the actual amount of gene flow reduction for a specific incompatibility level. In general, MtNI reduce gene flow stronger (weaker) than NNI when incompatibilities are recessive (dominant) (Figures 4, 5, Figure S3). The most pronounced differences occur if incompatibilities are purely recessive. Recessive NNI are well-known to have no effect on gene flow under weak migration because incompatibilities are masked in hybrids (Gavrilets, 1997). As explained in section Model D: nuclear-nuclear incompatibilities under diploid genetics, this is true for arbitrary incompatibility level and irrespective of whether incompatibilities are symmetric or asymmetric (Figure 4D). In contrast, recessive MtNI can have a strong effect on gene flow because female migrants have a reproductive value of $\frac{3+s_{2}}{3+3s_{2}}$. This is because mito-nuclear incompatibilities can only be fully masked in F1 hybrids of female migrants, but not in subsequent generations, as visible in the fitness graph (Figure S2). Accordingly, strong gene flow reductions occur for high levels of incompatibility, and the highest reductions of 33.3% under recessive MtNI occur in scenarios where only females migrate (Figure 4A).

## Discussion

We investigated the role of mito-nuclear incompatibilities on genetic divergence and speciation, and compared the results with models of nuclear-nuclear incompatibilities. Our main results are as follows. First, for haploid genetic systems, we found that gene flow reduction at an unlinked nuclear locus caused by MtNI is exactly the same as for NNI, but only if males and females migrate in equal number. Second, for models with diploid genetics, we found that MtNI reduces gene flow more strongly than NNI when incompatibilities are recessive, and less strongly when they are dominant. Third, the sex ratio among migrants has a significant effect on the results, but only in case of MtNI and not for NNI. For most scenarios analyzed, we found that gene flow reduction is stronger if females are predominantly the migrating sex, but weaker than NNI when males are predominantly the migrating sex.

These results generally support the view that mitochondria and other cytoplasmically inherited elements could promote nuclear genetic divergence and speciation. Most notably, we found for the diploid case that MtNI is more effective in reducing gene flow at a neutrally selected nuclear locus than NNI if genetic incompatibilities are recessive. This finding is noteworthy because nuclear-nuclear incompatibilities tend to be recessive early in the speciation process (Wu, 2001; Coyne and Orr, 2004; Wu and Ting, 2004). Hence, all other things being equal, MtNI is more effective than NNI to reduce gene flow and to select for postzygotic isolation in the crucial early stages of speciation.

Our analysis revealed a fundamental difference between mito-nuclear and nuclear-nuclear incompatibilities. For MtNI, we found that the sex ratio among migrants has a strong effect on the strength of gene flow reduction because males and females have different reproductive values in this case due to the exclusive inheritance of mitochondria through females. For NNI, however, the reproductive values are equal (so long as the fitness of hybrid genotypes are the same in males and females) and the sex ratio of migrants has no effect on gene flow. This observation may guide future empirical research. Our analysis of MtNI revealed the strongest gene flow reductions are for scenario in which only females migrate. We therefore hypothesize that MtNI is most effective in promoting speciation if females are the migrating sex (female migration hypothesis, FMH). There is some empirical support for this hypothesis. Parasitic wasps of the genus Nasonia are well-known for their mito-nuclear incompatibilities. Intriguingly, only female wasps migrate in these species, whereas males stay and mate at the site of emergence (Werren and Loehlin, 2009; Watt and Shuker, 2010).

Our theory may be tested by a comparative analysis similar to previous work on parapatric speciation. The reinforcement theory of speciation predicts that sym- or parapatric populations of incipient species show larger mate discrimination than allopatric populations, a phenomenon called reproductive character displacement (RCD) (Butlin, 1987). Coyne and Orr (1989, 1997) found RCD in *Drosophila* when they screened 171 pairs of different *Drosophila* populations. But RCD has also been found in several other taxa; for example, fishes and frogs (Noor, 1999). In order to test mitochondrial driven speciation, we suggest to reanalyze the existing *Drosophila* data, but to take into account migration patterns and mitochondrial evolution. The f*emale migration hypothesis* could be tested by comparing the rate of nuclear divergence with the percentage of females among migrants for species pairs with similar rates of mitochondrial genome substitutions. A positive correlation may be considered as indirect evidence for the FMH in the same way as RCD is indirect evidence for the reinforcement theory.

In the present study, we analyzed the effect of MtNI on gene flow between populations. Another important question is how these incompatibilities evolved in the first place. One hypothesis is that MtNI are caused by selective sweeps of certain mitochondrial haplotypes, and subsequent compensatory mutations in the nucleus (Rand et al., 2004; Oliveira et al., 2008; Raychoudhury et al., 2010). Obviously, high mutation rates of mitochondria would promote this process. There is evidence of considerable variation in rates and coevolution of mitochondrial and interacting nuclear genes in many taxa (Arnold, 1993; Rand et al., 2004; Connallon et al., 2018; Yan et al., 2018). However, nuclear-mitochondrial incompatibilities are also common in plants (Fishman and Sweigart, 2018), which generally do not show an accelerated mitochondrial mutation rate. Cytoplasmically inherited bacteria, such as *Wolbachia*, may play a complementary role in this process in arthropods, where this intracellular bacterium is common. Once they enter a new host species, they spread rapidly up to high frequencies and cause sweeps of mitochondrial haplotypes due to genetic hitchhiking (Oliveira et al., 2008; Raychoudhury et al., 2010). This makes *Wolbachia* a potentially important driver for mitochondrial genome evolution in general, and for the evolution of MtNI in particular. From the theoretical perspective, these results raise the question how gene flow is reduced in the presence of both *Wolbachia* and nuclear-mitochondrial incompatibilities. Analyzing the joined dynamics may be promising topic for future research.

Another important question relates to the stability of genetic divergence in the presence of migration. It is well-known that genetic divergence between populations is lost if local selection is weak in comparison to migration. For instance, the well-studied Dobzhansky-Muller incompatibilities can only persist in the presence of migration if they are dominant or co-dominant, but genetic divergence is lost if the incompatibility is recessive (Bengtsson, 1985; Gavrilets, 1997; Kobayashi and Telschow, 2008; Bank et al., 2012; Telschow et al., 2014). A preliminary analysis of MtNI shows qualitatively similar results (results not shown). An important direction for future research may be to analyze whether additional genetic factors such as *Wolbachia* can stabilize or destabilize the genetic divergence of MtNI, as it has shown previously for *Wolbachia* and Dobzhansky-Muller incompatibilities (Telschow et al., 2014). A third area for exploration is the role of linkage between incompatible loci and neutral loci on effective migration rates and gene flow. This is of particular interest because of differences in nuclear and mitochondrial flow in hybrid zones (Arnold, 1993).

In conclusion, our theoretical analysis suggests that mito-nuclear incompatibilities are as effective or even more effective in reducing gene flow than nuclear-nuclear incompatibilities depending on the life history of a species (e.g., female specific migration). These results encourage further examination on the role of mitochondria and other cytoplasmically inherited genetic elements on genetic divergence and speciation and make specific predictions. For example, MtNI should be more pronounced in species where females are the dispersing sex.

## Author Contributions

AT, YK, JG, and JW contributed conception and design of the study. AT and YK performed the mathematical analysis. AT wrote the first draft of the manuscript. YK wrote sections of the manuscript. All authors contributed to manuscript revision, read, and approved the submitted version.

### Conflict of Interest Statement

The authors declare that the research was conducted in the absence of any commercial or financial relationships that could be construed as a potential conflict of interest.
