# Supplementary material for: Genetic Incompatibilities Between Mitochondria and Nuclear Genes: Effect on Gene Flow and Speciation
Source: Front Genet. 2019 Feb 13;10:62. doi: 10.3389/fgene.2019.00062 (PMC6396729; doi:10.3389/fgene.2019.00062)
Supplement: Supplementary file 1 [file Data_Sheet_1.pdf]

Supplementary Information

Genetic incompatibilities between mitochondria and nuclear genes: effect on gene flow and speciation

Authors

Arndt Telschow[*,1], Jürgen Gadau[2], John H. Werren[3], Yutaka Kobayashi[4]

[1] Institute for Environmental Systems Research, Osnabruck University, Osnabrück, Germany

[2] Institute for Evolution and Biodiversity, Westfalian Wilhelms-University, Münster, Germany

[3] Department of Biology, Rochester University, USA

[4] Kochi University of Technology, Japan

[*] Corresponding author: atelschow@uni-osnabrueck.de

S1 Recursion equations used for numerical analysis

Below, we give for all four models a formal description of the recursion equations that are used for the numerical analysis of the effective migration rate (Fig. S4). The recursion equations describe the change in allele frequency over time. For a given parameter set, the effective migration rate is calculated by substituting the allele frequencies at the marker locus in formula (1). We assume that the neutral marker locus has two alleles, M_1 and M_2, and denote the frequency of allele M_2 in the island population in generation t by $p(t)$. The frequency q of M_2 in the mainland population does not affect the effective migration rate as far as it is constant and the limit is taken as $t \to \infty$ (Kobayashi et al., 2008). Therefore, we assume that the mainland population is fixed for allele M_2 (q=1).

Model A: The haploid model with mito-nuclear incompatibility

Let $F_t(mt_iN_jM_k)$ and $G_t(mt_iN_jM_k)$ be the frequencies females and males, respectively, of genotype $mt_iN_jM_k$ in the island population at the beginning of generation t. Note that the frequencies are here defined as fractions relative to the entire island population. It holds that

$$\text{(A.1)} \qquad \sum_{i=1}^{2}\sum_{j=1}^{2}\sum_{k=1}^{2} F_t(mt_iN_jM_k) = \sum_{i=1}^{2}\sum_{j=1}^{2}\sum_{k=1}^{2} G_t(mt_iN_jM_k) = \frac{1}{2}.$$

The even sex ratio is maintained because the primary sex ratio is even and both sexes suffer equally from genetic incompatibilities. In the following, we derive recursion equations that describe the allele frequency change between subsequent generations.

Let K and L be the size of the island population and the number of immigrants per generation, respectively. Let also e_f and e_m be the emigration rates of females and males, respectively, from the island population. By definition, the immigration rate m is given by

$$\text{(A.2)} \qquad m = \frac{L}{\left(\frac{K}{2}\right)(1-e_f)+\left(\frac{K}{2}\right)(1-e_m)+L} = \frac{L}{K\left(1-\frac{e_f+e_m}{2}\right)+L}.$$

We assume that the total number of emigrants is equal to the number of immigrants, so that

$$\text{(A.3)} \qquad K\left(\frac{e_f+e_m}{2}\right) = L.$$

Substituting (A.3) into (A.2) yields

$$\text{(A.4)} \qquad m = \frac{e_f+e_m}{2}.$$

Further, we assume that the number of emigrating females (males) is equal to the number of immigrating females (males), so that

$$\text{(A.5)} \qquad \left(\frac{K}{2}\right)e_f = Lm_f,$$

(A.6) $\left(\frac{K}{2}\right) e_m = L(1 - m_f).$

Combining equations (A.3)-(A.6) yields

(A.7) $e_f = 2mm_f,$

(A.8) $e_m = 2m(1 - m_f).$

Let $F_t^+(mt_iN_iM_i)$ and $G_t^+(mt_iN_iM_i)$ be the frequencies of females and males, respectively, of genotype $mt_iN_iM_i$ in the island population immediately after migration in generation *t*. It holds that

(A.9) $$F_t^+(mt_iN_jM_k) = \frac{(1-e_f)KF_t(mt_iN_iM_k)+Lm_f\delta_{2i}\delta_{2j}\delta_{2k}}{K\left(1-\frac{e_f+e_m}{2}\right)+L},$$

(A.10) $$G_t^+(mt_iN_jM_k) = \frac{(1-e_m)KG_t(mt_iN_jM_k)+L(1-m_f)\delta_{2i}\delta_{2j}\delta_{2k}}{K\left(1-\frac{e_f+e_m}{2}\right)+L},$$

where δ_{ij} is the Kronecker's delta. Substituting (A.3) and (A.4) into (A.9)-(A.10) yields

(A.11) $F_t^+(mt_iN_jM_k) = (1 - 2mm_f)F_t(mt_iN_jM_k) + mm_f\delta_{2i}\delta_{2j}\delta_{2k},$

(A.12) $G_t^+(mt_iN_jM_k) = \left(1 - 2m(1 - m_f)\right) G_t(mt_iN_jM_k) + m(1 - m_f)\delta_{2i}\delta_{2j}\delta_{2k}.$

Summing up (A.11) and (A.12) over all genotypes shows that migration does not affect the sex ratio. Note that the frequency of $mt_iN_jM_k$ among females (males) after migration is given by $\frac{F_t^+(mt_iN_jM_k)}{\frac{1}{2}} = 2F_t^+(mt_iN_jM_k)$ $(2G_t^+(mt_iN_jM_k))$. Therefore, the frequency of matings between $mt_iN_jM_k$ females and $mt_uN_vM_w$ males is given by $4F_t^+(mt_iN_jM_k)G_t^+(mt_uN_vM_w)$. Therefore, from Table 1, genotype frequencies in the next generations are given by

(A.13) $$F_{t+1}(mt_aN_bM_c) = G_{t+1}(mt_aN_bM_c) = \frac{1}{2T}\sum_{j=1}^{2}\sum_{k=1}^{2}\sum_{u=1}^{2}\sum_{v=1}^{2}\sum_{w=1}^{2} 4F_t^+(mt_aN_jM_k)G_t^+(mt_uN_vM_w)\left(\frac{\delta_{bj}+\delta_{bv}}{2}\right)\left(\frac{\delta_{ck}+\delta_{cw}}{2}\right)\left(1 - s_a(1 - \delta_{ab})\right),$$

where

$$T = \sum_{a=1}^{2}\sum_{b=1}^{2}\sum_{c=1}^{2}\sum_{j=1}^{2}\sum_{k=1}^{2}\sum_{u=1}^{2}\sum_{v=1}^{2}\sum_{w=1}^{2} 4F_t^+(mt_aN_jM_k)G_t^+(mt_uN_vM_w)\left(\frac{\delta_{bj}+\delta_{bv}}{2}\right)\left(\frac{\delta_{ck}+\delta_{cw}}{2}\right)\left(1 - s_a(1 - \delta_{ab})\right).$$

We can compute genotype frequencies in any generation by iterating (A.11)-(A.13). In particular, the frequency of allele M_2 at generation *t* is given by

(A.14) $p(t) = \sum_{i=1}^{2}\sum_{j=1}^{2}\left(F_t(mt_iN_jM_2) + G_t(mt_iN_jM_2)\right).$

For the numerical analysis, we used the initial condition that M_2 is absent in the island population, $F_0(mt_iN_jM_k) = G_0(mt_iN_jM_k) = \frac{\delta_{1i}\delta_{1j}\delta_{1k}}{2}$, iterated equations (A.11)-(A.13) a fixed number of times, and calculated *p*(*t*). The effective migration rate was calculated by substituting *p*(*t*) in formulae (1). As a result, we found that the numerical estimates of the effective migration rate converge to the analytical formula shown in equation (14) if the limit is taken for $t \to \infty$ (Fig. S4).

Model B: The haploid model with nuclear-nuclear incompatibility

For *Model B*, we do not have to distinguish males and females, and assume that the organisms are hermaphrodites. Let $H_t(A_iB_jM_k)$ be the frequency of genotype $A_iB_jM_k$ in the island population at the beginning of generation *t*, where M_i denotes the alleles at the marker locus. The allele frequencies after migration are given by

(A.15) $H_t^+(A_iB_jM_k) = (1-m)H_t(A_iB_jM_k) + m\delta_{2i}\delta_{2j}\delta_{2k},$

given that the emigration rate is equal to the immigration rate *m*. From Table 2, genotype frequencies in the next generation are given by

(A.16) $H_{t+1}(A_aB_bM_c) =$
$\frac{1}{T}\sum_{i=1}^{2}\sum_{j=1}^{2}\sum_{k=1}^{2}\sum_{u=1}^{2}\sum_{v=1}^{2}\sum_{w=1}^{2} H_t^+(A_iB_jM_k)H_t^+(A_uB_vM_w)\left(\frac{\delta_{ai}+\delta_{au}}{2}\right)\left(\frac{\delta_{bj}+\delta_{bv}}{2}\right)\left(\frac{\delta_{ck}+\delta_{cw}}{2}\right)(1 -$
$s_a(1-\delta_{ab})),$

where $s_1 = s_B$, $s_2 = s_A$ and

$T =$
$\sum_{a=1}^{2}\sum_{b=1}^{2}\sum_{i=1}^{2}\sum_{j=1}^{2}\sum_{k=1}^{2}\sum_{u=1}^{2}\sum_{v=1}^{2}\sum_{w=1}^{2} H_t^+(A_iB_jM_k)H_t^+(A_uB_vM_w)\left(\frac{\delta_{ai}+\delta_{au}}{2}\right)\left(\frac{\delta_{bj}+\delta_{bv}}{2}\right)(1 -$
$s_a(1-\delta_{ab})).$

The initial condition is given by $H_0(A_iB_jM_k) = \delta_{1i}\delta_{1j}\delta_{1k}$ and the frequency of allele M_2 by

(A.17) $p(t) = \sum_{i=1}^{2}\sum_{j=1}^{2} H_t(A_iB_jM_2).$

As above, the effective migration rate was calculated by substituting *p*(*t*) in formulae (1). As a result, we found that the numerical estimates of the effective migration rate converge to the analytical formula shown in equation (22) in the limit $t \to \infty$ (Fig. S4).

Model C: The diploid model with mito-nuclear incompatibility

For convenience, we let N_jN_k (M_lM_n) denote the genotype of an individual who inherited N_j (M_l) from its mother and N_k (M_n) from its father. Let $F_t(mt_iN_jN_kM_lM_n)$ and $G_t(mt_iN_jN_kM_lM_n)$ be the frequencies of females and males, respectively, of genotype $mt_iN_jN_kM_lM_n$ in the island population in generation *t*. Following the same arguments as for *Model A*, we find that the genotype frequencies after migration are given by

(A.18) $$F_t^+(mt_iN_jN_kM_lM_n) = (1-2mm_f)F_t(mt_iN_jN_kM_lM_n) + mm_f\delta_{2i}\delta_{2j}\delta_{2k}\delta_{2l}\delta_{2n},$$

(A.19) $$G_t^+(mt_iN_jN_kM_lM_n) = \left(1-2m(1-m_f)\right)G_t(mt_iN_jN_kM_lM_n) + m(1-m_f)\delta_{2i}\delta_{2j}\delta_{2k}\delta_{2l}\delta_{2n}.$$

From Table 3, genotype frequencies in the next generation are given by

(A.20) $$F_{t+1}(mt_aN_bN_cM_dM_e) = G_{t+1}(mt_aN_bN_cM_dM_e)$$
$$= \frac{1}{2T}\sum_{j=1}^{2}\sum_{k=1}^{2}\sum_{l=1}^{2}\sum_{n=1}^{2}\sum_{u=1}^{2}\sum_{v=1}^{2}\sum_{w=1}^{2}\sum_{x=1}^{2}\sum_{y=1}^{2} 4F_t^+(mt_aN_jN_kM_lM_n)G_t^+(mt_uN_vN_wM_xM_y)\cdot \left(\frac{\delta_{bj}+\delta_{bk}}{2}\right)\left(\frac{\delta_{cv}+\delta_{cw}}{2}\right)\left(\frac{\delta_{dl}+\delta_{dn}}{2}\right)\left(\frac{\delta_{ex}+\delta_{ey}}{2}\right)[1 - h_as_a\{(1-\delta_{ab})\delta_{ac}+(1-\delta_{ac})\delta_{ab}\}],$$

where $T =$

$$\sum_{a=1}^{2}\sum_{b=1}^{2}\sum_{c=1}^{2}\sum_{d=1}^{2}\sum_{e=1}^{2}\sum_{j=1}^{2}\sum_{k=1}^{2}\sum_{l=1}^{2}\sum_{n=1}^{2}\sum_{u=1}^{2}\sum_{v=1}^{2}\sum_{w=1}^{2}\sum_{x=1}^{2}\sum_{y=1}^{2} 4F_t^+(mt_aN_jN_kM_lM_n)G_t^+(mt_uN_vN_wM_xM_y) \left(\frac{\delta_{bj}+\delta_{bk}}{2}\right)\left(\frac{\delta_{cv}+\delta_{cw}}{2}\right)\left(\frac{\delta_{dl}+\delta_{dn}}{2}\right)\left(\frac{\delta_{ex}+\delta_{ey}}{2}\right)[1-h_as_a\{(1-\delta_{ab})\delta_{ac}+(1-\delta_{ac})\delta_{ab}\}]\left(1-s_a(1-\delta_{ab})(1-\delta_{ac})\right).$$

The initial condition is given by $F_0(mt_iN_jN_kM_lM_n) = G_0(mt_iN_jN_kM_lM_n) = \frac{\delta_{1i}\delta_{1j}\delta_{1k}\delta_{1l}\delta_{1n}}{2}$ and the frequency of the allele M_2 by

(A.21) $$p(t) = \sum_{i=1}^{2}\sum_{j=1}^{2}\sum_{k=1}^{2}\sum_{l=1}^{2}\sum_{n=1}^{2}\left(F_t(mt_iN_jN_kM_lM_n) + G_t(mt_iN_jN_kM_lM_n)\right)\left(\frac{\delta_{2l}+\delta_{2n}}{2}\right).$$

As above, the effective migration rate was calculated by substituting $p(t)$ in formulae (1). As a result, we found that the numerical estimates of the effective migration rate converge to the analytical formula shown in equation (35) in the limit $t \to \infty$ (Fig. S4).

Model D-1: The diploid model with asymmetric nuclear-nuclear incompatibility

As in *Model B*, we assume that the organisms under consideration are hermaphrodites. As in *Model C*, we let A_iA_j denote the genotype of an individual who inherited A_i from its "mother" (e.g., the individual who contributed an ovule) and A_j from its "father" (e.g., the individual who contributed pollen). The same notation applies to the second incompatibility locus and the neutral marker locus. Let $H_t(A_iA_jB_kB_lM_nM_r)$ denote the frequency of genotype $A_iA_jB_kB_lM_nM_r$ at the beginning of generation *t*. Then, the genotype frequencies after migration are given by

$$\text{(A.22)} \qquad H_t^+(A_iA_jB_kB_lM_nM_r) = (1-m)H_t(A_iA_jB_kB_lM_nM_r) + m\delta_{2i}\delta_{2j}\delta_{2k}\delta_{2l}\delta_{2n}\delta_{2r}.$$

From Table 4, genotype frequencies in the next generation are given by

$$\text{(A.23)} \qquad H_{t+1}(A_aA_bB_cB_dM_eM_f) = \frac{1}{T}\sum_{i=1}^{2}\sum_{j=1}^{2}\sum_{k=1}^{2}\sum_{l=1}^{2}\sum_{n=1}^{2}\sum_{r=1}^{2}\sum_{u=1}^{2}\sum_{v=1}^{2}\sum_{w=1}^{2}\sum_{x=1}^{2}\sum_{y=1}^{2}\sum_{z=1}^{2} H_t^+(A_iA_jB_kB_lM_nM_r)$$

$$H_t^+(A_uA_vB_wB_xM_yM_z)\left(\frac{\delta_{ai}+\delta_{aj}}{2}\right)\left(\frac{\delta_{bu}+\delta_{bv}}{2}\right)\left(\frac{\delta_{ck}+\delta_{cl}}{2}\right)\left(\frac{\delta_{dw}+\delta_{dx}}{2}\right)\left(\frac{\delta_{en}+\delta_{er}}{2}\right)\left(\frac{\delta_{fy}+\delta_{fz}}{2}\right)$$

$$(1-s_A\delta_{2a}\delta_{2b}\delta_{1c}\delta_{1d})(1-h_As_A(1-\delta_{ab}\delta_{cd})(1-\delta_{1a}\delta_{1b})(1-\delta_{2c}\delta_{2d})).$$

where

$$T = \sum_{a=1}^{2}\sum_{b=1}^{2}\sum_{c=1}^{2}\sum_{d=1}^{2}\sum_{e=1}^{2}\sum_{f=1}^{2}\sum_{i=1}^{2}\sum_{j=1}^{2}\sum_{k=1}^{2}\sum_{l=1}^{2}\sum_{n=1}^{2}\sum_{r=1}^{2}\sum_{u=1}^{2}\sum_{v=1}^{2}\sum_{w=1}^{2}\sum_{x=1}^{2}\sum_{y=1}^{2}\sum_{z=1}^{2}$$

$$H_t^+(A_iA_jB_kB_lM_nM_r)H_t^+(A_uA_vB_wB_xM_yM_z)$$

$$\left(\frac{\delta_{ai}+\delta_{aj}}{2}\right)\left(\frac{\delta_{bu}+\delta_{bv}}{2}\right)\left(\frac{\delta_{ck}+\delta_{cl}}{2}\right)\left(\frac{\delta_{dw}+\delta_{dx}}{2}\right)\left(\frac{\delta_{en}+\delta_{er}}{2}\right)\left(\frac{\delta_{fy}+\delta_{fz}}{2}\right)$$

$$(1-s_A\delta_{2a}\delta_{2b}\delta_{1c}\delta_{1d})(1-h_As_A(1-\delta_{ab}\delta_{cd})(1-\delta_{1a}\delta_{1b})(1-\delta_{2c}\delta_{2d})).$$

The initial condition is given by $H_0(A_iA_jB_kB_lM_nM_r) = \delta_{1i}\delta_{1j}\delta_{1k}\delta_{1l}\delta_{1n}\delta_{1r}$ and the frequency of allele M_2 at generation *t* by

$$\text{(A.24)} \qquad p(t) = \sum_{i=1}^{2}\sum_{j=1}^{2}\sum_{k=1}^{2}\sum_{l=1}^{2}\sum_{n=1}^{2}\sum_{r=1}^{2} H_t(A_iA_jB_kB_lM_nM_r)\left(\frac{\delta_{2n}+\delta_{2r}}{2}\right).$$

The convergence of equation (1) to equation (36) can be confirmed as we did for *Model A*.

Model D-2: The diploid model with symmetric nuclear-nuclear incompatibility

In the case of symmetric nuclear-nuclear incompatibility, eq. (A.21) still holds for the migration phase. For the mating phase, from Table 5, we have

(A.25) $$H_{t+1}(A_aA_bB_cB_dM_eM_f) = \frac{1}{T}\sum_{i=1}^{2}\sum_{j=1}^{2}\sum_{k=1}^{2}\sum_{l=1}^{2}\sum_{n=1}^{2}\sum_{r=1}^{2}\sum_{u=1}^{2}\sum_{v=1}^{2}\sum_{w=1}^{2}\sum_{x=1}^{2}\sum_{y=1}^{2}\sum_{z=1}^{2} H_t^+(A_iA_jB_kB_lM_nM_r) H_t^+(A_uA_vB_wB_xM_yM_z)\left(\frac{\delta_{ai}+\delta_{aj}}{2}\right)\left(\frac{\delta_{bu}+\delta_{bv}}{2}\right)\left(\frac{\delta_{ck}+\delta_{cl}}{2}\right)\left(\frac{\delta_{dw}+\delta_{dx}}{2}\right)\left(\frac{\delta_{en}+\delta_{er}}{2}\right)\left(\frac{\delta_{fy}+\delta_{fz}}{2}\right) \left(1-s\delta_{ab}\delta_{cd}(1-\delta_{ac})\right)\left(1-hs(1-\delta_{ab}\delta_{cd})\right),$$

where

$$T = \sum_{a=1}^{2}\sum_{b=1}^{2}\sum_{c=1}^{2}\sum_{d=1}^{2}\sum_{e=1}^{2}\sum_{f=1}^{2}\sum_{i=1}^{2}\sum_{j=1}^{2}\sum_{k=1}^{2}\sum_{l=1}^{2}\sum_{n=1}^{2}\sum_{r=1}^{2}\sum_{u=1}^{2}\sum_{v=1}^{2}\sum_{w=1}^{2}\sum_{x=1}^{2}\sum_{y=1}^{2}\sum_{z=1}^{2} H_t^+(A_iA_jB_kB_lM_nM_r)H_t^+(A_uA_vB_wB_xM_yM_z)$$

$$\left(\frac{\delta_{ai}+\delta_{aj}}{2}\right)\left(\frac{\delta_{bu}+\delta_{bv}}{2}\right)\left(\frac{\delta_{ck}+\delta_{cl}}{2}\right)\left(\frac{\delta_{dw}+\delta_{dx}}{2}\right)\left(\frac{\delta_{en}+\delta_{er}}{2}\right)\left(\frac{\delta_{fy}+\delta_{fz}}{2}\right)\left(1-s\delta_{ab}\delta_{cd}(1-\delta_{ac})\right)\left(1-hs(1-\delta_{ab}\delta_{cd})\right).$$

The initial condition is given by $H_0(A_iA_jB_kB_lM_nM_r) = \delta_{1i}\delta_{1j}\delta_{1k}\delta_{1l}\delta_{1n}\delta_{1r}$ and the frequency of allele M_2 by equation (A.24). The convergence of equation (1) can be checked in the same way as we did for *Model A*.

S2 Supplementary Figures

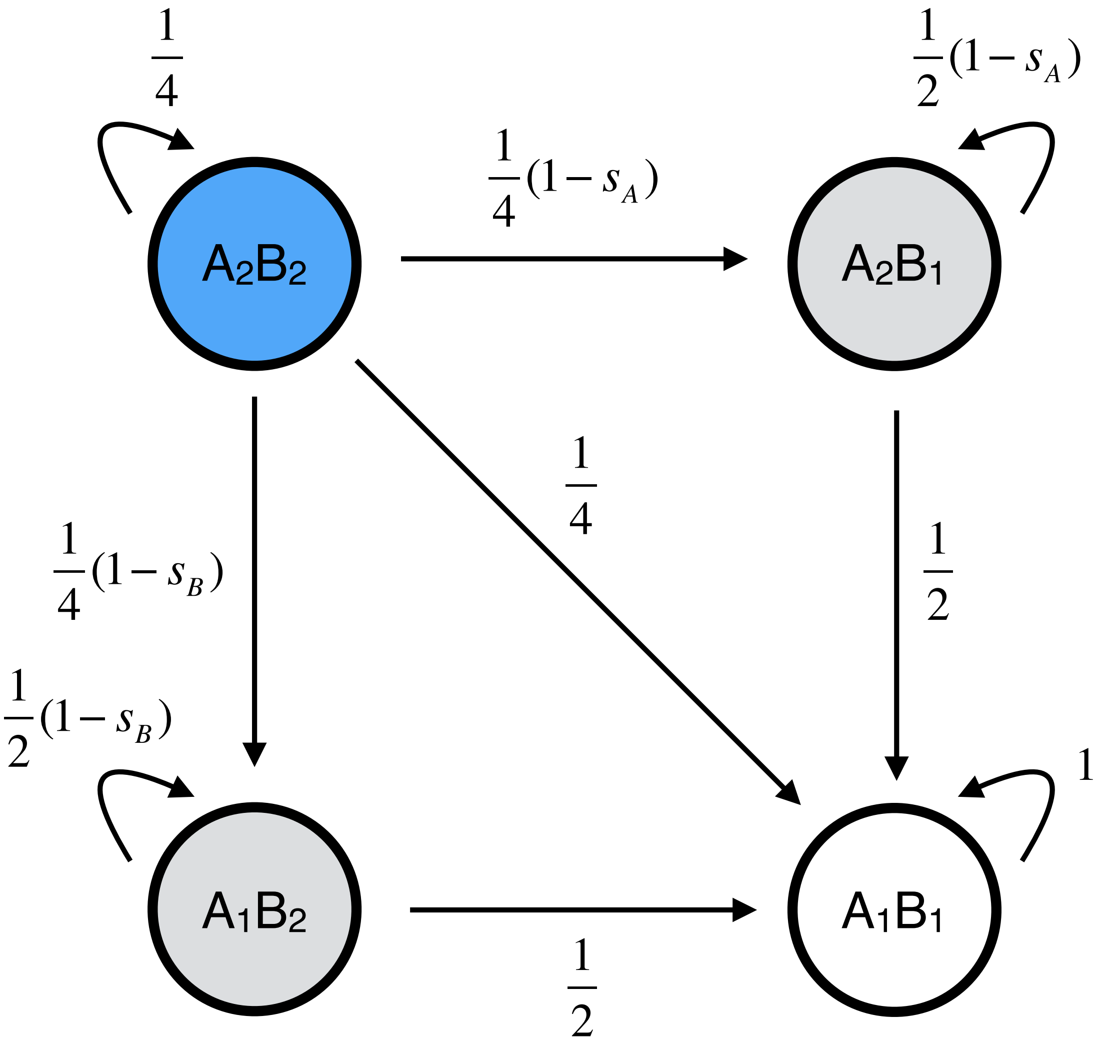

Figure S1. Fitness graph for haploid NNI. Shown is the fitness graph for *Model B* (nuclear-nuclear incompatibilities, haploid genetics). There are four genetic classes. Class transitions are indicated by black arrows and attached weighting factors. The graph describes gene flow of rare male and female migrants of genotype A_2B_2 in an island population consisting of mostly A_1B_1 individuals. Blue indicates the migrant genotypes, gray the hybrid genotypes, and white the resident genotypes. Gene flow is measured at a neutrally selected nuclear marker locus, which is not shown in the graph (Methods).

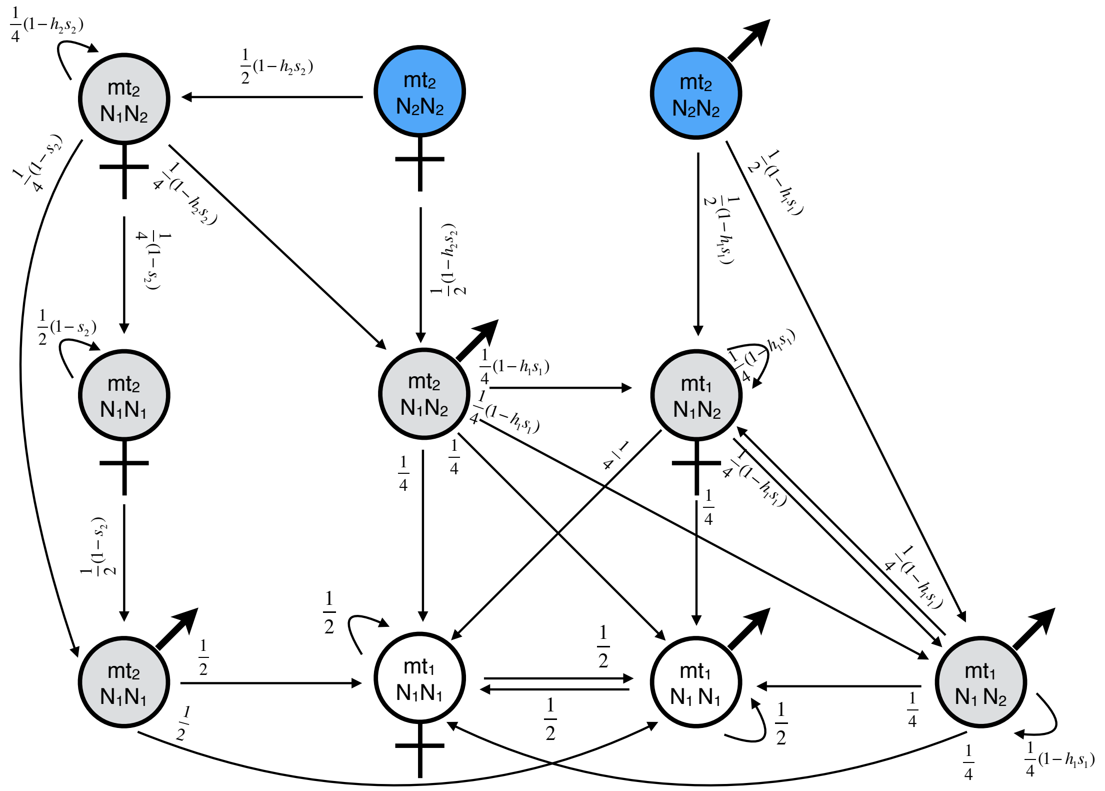

Figure S2. Fitness graph for diploid MtNI. Shown is the fitness graph for *Model C* (mito-nuclear incompatibilities, diploid genetics). There are ten genetic classes. Class transitions are indicated by black arrows and attached weighting factors. The graph describes gene flow of rare male and female migrants of genotype $mt_2N_2N_2$ in an island population consisting of mostly $mt_1N_1N_1$ individuals. Blue indicates the migrant genotypes, gray the hybrid genotypes, and white the resident genotypes. Gene flow is measured at a neutrally selected nuclear marker locus, which is not shown in the graph (Methods).

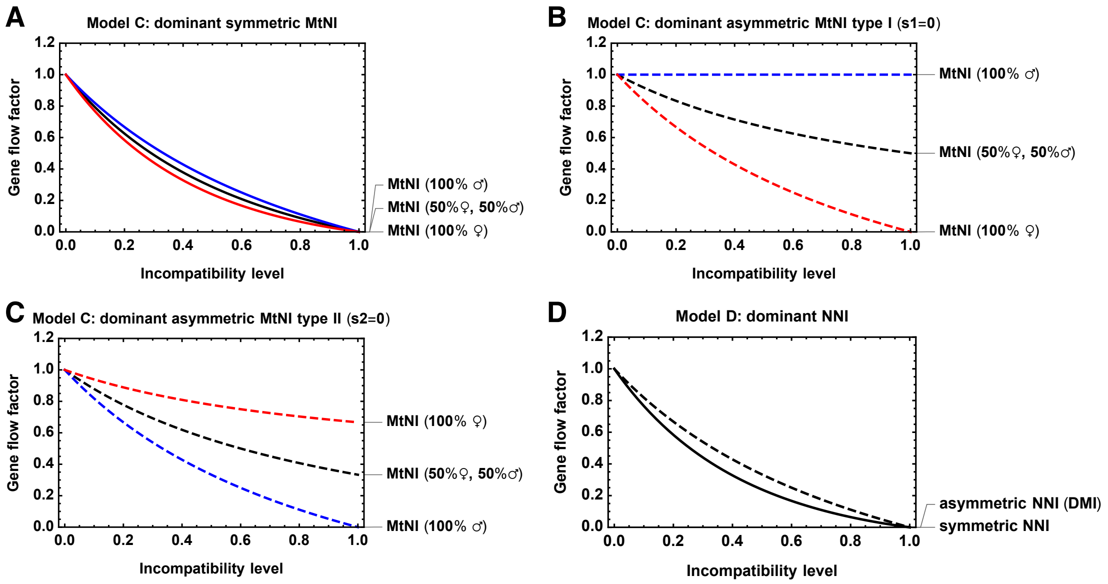

Figure S3. Gene flow reduction for models with diploid genetics. Shown is the gene flow factor for an unlinked neutral locus as a function of the level of incompatibility. **(A)** *Model C* with dominant symmetric MtNI and varying incompatibility level s_1=s_2. **(B)** *Model C* with dominant asymmetric MtNI of type I (s_1=0) and varying incompatibility level s_2. **(C)** *Model C* with dominant asymmetric MtNI of type II (s_2=0) and varying incompatibility level s_1. **(D)** *Model D* with dominant symmetric (s_A= s_B) and asymmetric (s_B=0) NNI and varying incompatibility level s_A. Parameters: h_1=h_2=h_A=h_B=1.

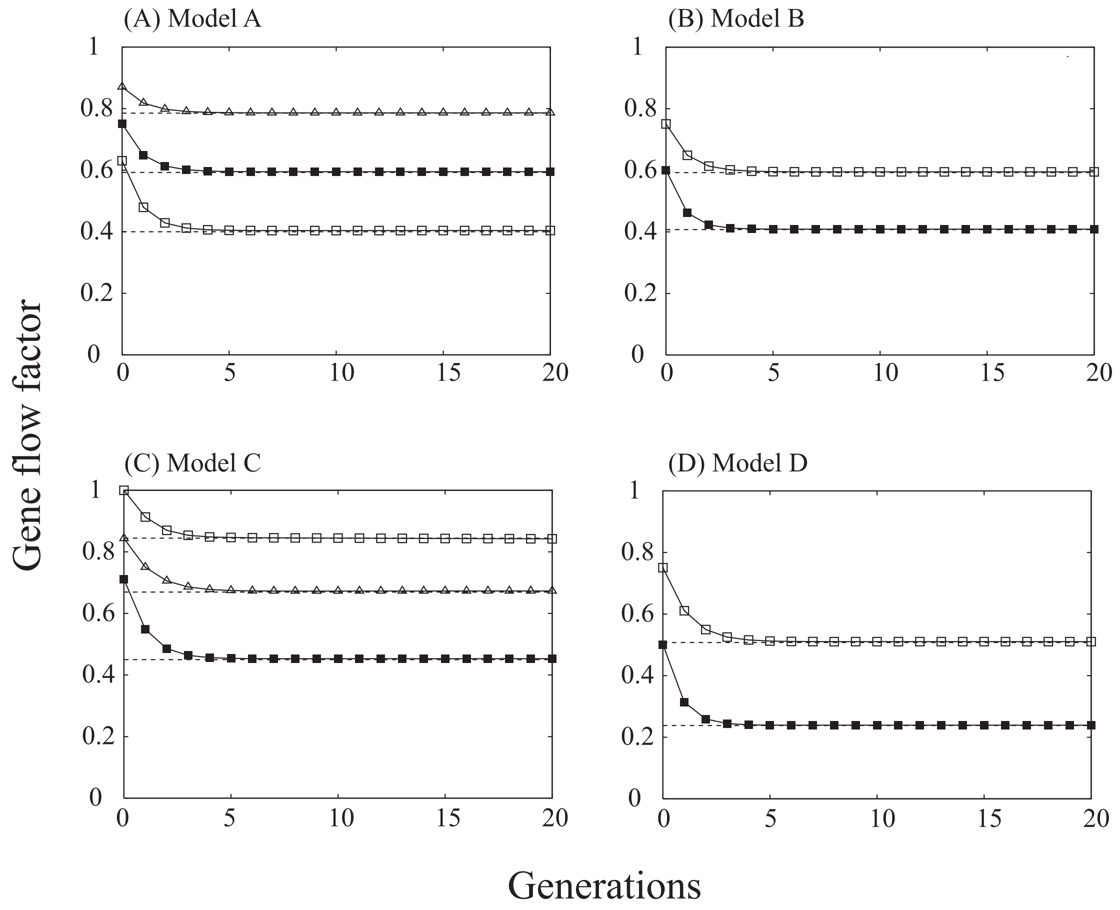

Figure S4. Gene flow factor: numerical calculations versus analytical formulae. The figure shows that the numerical estimates of the gene flow factor approximate the derived analytical formula well. Numerical estimates were calculated by numerical iteration of recursion equations (see SI), and subsequent substitution of the marker allele frequencies in formula (1). Solid lines indicate numerical estimates of the gene flow factor as a function of the generation, t, and dashed lines the results of the corresponding analytical formulae. **(A)** Model A with s_1=0.2, s_2=0.8, m=0.001, m_f=0.9 (open squares), m_f=0.5 (filled squares), m_f=0.1 (open triangles). **(B)** Model B with m=0.001, s_A=s_B=0.8 (open squares), s_A=0.8, s_B=0.2 (filled squares). **(C)** Model C with m=0.001, m_f=0.7, s_1=s_2=0.5, h_1=h_2=0 (open squares), s_1=0.5, s_2=0.2, h_1=h_2=1 (filled squares), s_1=0.5, s_2=0.2, h_1=0.3, h_2=0.8 (open triangles). **(D)** Model D with m=0.001, s_A=s_B=0.5, h_A=h_B=0.5 (open squares), h_A= h_B=1 (filled squares).
